# From developmental theory to effective training: long-term and transfer effects of promoting the quantity–to–number word linkage in first-graders at risk for mathematical difficulties

**DOI:** 10.3389/fpsyg.2024.1380036

**Published:** 2024-08-20

**Authors:** Marco Ennemoser, Daniel Sinner, Linda Nguyen, Kristin Krajewski

**Affiliations:** ^1^Faculty of Participation Science, University of Education Ludwigsburg, Ludwigsburg, Germany; ^2^Institute for Psychology, Justus Liebig University Gießen, Giessen, Germany; ^3^Institute for Psychology, University of Education Ludwigsburg, Ludwigsburg, Germany

**Keywords:** number sense, prevention, developmental dyscalculia, approximate number system, math intervention, math development, QNL model, numerical vocabulary development

## Abstract

**Introduction:**

The model of quantity–to–number word linkage (*QNL model*) identifies relevant milestones in the process of early numerical acquisition and describes a developmental sequence that can guide the fostering of foundational mathematical abilities in at-risk children. While there is substantial evidence for the predictive value of the quantity-number competencies (QNC) described by the model, evidence supporting the preventive potential of interventions targeting these QNC is so far largely restricted to short-term effects. Findings regarding their long-term preventive impact, especially in terms of transfer to mathematical school achievement, are still limited. This quasi-experimental study aimed to address this gap by evaluating the long-term transfer effects of an intervention program that is strictly derived from the QNL model of mathematical development [QNL training; in German “Mengen, zählen, Zahlen” (MZZ)].

**Methods:**

We assessed the quantity-number competencies of 575 first-graders and identified 119 of them as being at risk for mathematical learning difficulties, who were then assigned to three experimental conditions. Sixty one children received 12 sessions of the QNL training, while 30 underwent training in inductive reasoning. Another 28 children served as a control group, receiving no specific intervention.

**Results and Discussion:**

Multi-level analyses confirmed both significant short-and long-term effects in the specifically trained quantity–number competencies as well as transfer effects on subsequent mathematical school achievement. In accordance with previous findings, transfer effects of the QNL training on mathematical school achievement were not yet evident immediately after the intervention but turned out to be significant after a delay of 6 months and remained stable even 15 months after training. Effect sizes ranged from *d* = 0.32 to *d* = 1.12. These findings both underscore the preventive potential of interventions that are strictly driven by developmental theory and, conversely, support the theoretical assumptions of the QNL model.

## 1 Introduction

### 1.1 Perspectives on the prevention of mathematical learning difficulties

One of the most important challenges in research on developmental dyscalculia is to find effective means of prevention. However, as noted by Szücs and Goswami (2013), many theoretical approaches in the field provide a rather pessimistic view of the prospects for achieving this goal, in that they suggest that children with developmental dyscalculia are lacking a cognitive core module critical to understanding mathematics, i.e., they display a neurological dysfunction (Shalev, 2004; Kucian et al., 2006; Mussolin et al., 2010). Those authors point out that this notion “may suggest to educators an irreversible condition” (p. 36). In the current paper we take a more optimistic position. Based on the *developmental model of quantity–to–number word linkage* (*QNL model*) (Krajewski, 2008; see Krajewski and Schneider, 2009a,b), we suggest that what is often labeled as developmental dyscalculia might more accurately be described as a delay in numerical development rather than a deficient cognitive module. Consequently, while we also use the term “developmental dyscalculia” to stay aligned with established clinical and educational terminology, we prefer to describe persistent challenges using the term “mathematical difficulties.” Unlike dyscalculia, this term does not imply assumptions on an immutable neurological deficit but rather emphasizes developmental gaps that can potentially be closed through specific training.

In the following section, we will give a brief description of the underlying model of early mathematical development presumed here and will suggest that effective prevention programs should address the particular levels of competence described in the model. Furthermore, we will report findings on the predictive value of the corresponding *quantity–number competencies* (QNC) and provide a discussion of existing studies on the effectiveness of early interventions for children with low mathematical achievement or at-risk children, respectively.

A particular focus will be on methodological limitations of the available studies, limitations that lead to the notion that the most important questions regarding the effectiveness of dyscalculia prevention programs remain unanswered, although most available studies report some kind of significant effect on numerical abilities. To overcome this unsatisfactory situation, we will make two suggestions for future research on the prevention and early intervention in math difficulties or dyscalculia:

(1) The content of a training program should be derived strictly based on a developmental theory able to explain why and how a particular training approach should lead to (long-term) improvement in subsequent school achievement in mathematics; and
(2) Research on the prevention of math difficulties should more seriously address some (well-known) methodological challenges that have yet to be met by evaluation studies, in order to provide more reliable evidence about what works (and does not), why, and how.

On the basis of these two suggestions, we will introduce a training program in quantity-to-number-word linkage (*QNL*) “Mengen, zählen, Zahlen” (Quantities, counting, numbers) (Krajewski et al., 2007) based on the developmental QNL model. Finally, we will report the results of a longitudinal intervention study on the effectiveness of this training program, through which we tried to address the methodological challenges that we will have discussed.

### 1.2 Developmental model of quantity–to–number word linkage (QNL model)

#### 1.2.1 Key principle and core assumptions of the QNL model

*Principle of conservative competence attribution*. The model of quantity–to–number word linkage (Krajewski, 2008; for English-language discussion, see also Krajewski and Schneider, 2009a,b, and the meta-analysis by Lin and Powell, 2023) describes numerical development (that is, the development of quantity–number competencies) from birth through to primary school. A key distinction of the QNL model from other approaches is the *principle of conservative competence attribution*. This principle posits that any observable mathematical “output” in a child’s behavior should be interpreted conservatively to avoid overestimating the child’s numerical abilities. For example, unlike other researchers in the field (e.g., Fuson, 1988; von Aster and Shalev, 2007), the QNL model suggests that merely saying a single number word does not necessarily imply that the child fully understands its meaning (i.e., that the word precisely represents a specific quantity). The QNL model attributes observed performances, such as reciting number words backwards, only to competencies deemed necessary, essential, and sufficient to produce that performance.

This conservative attribution allows for a more sensitive detection of potential hurdles that make some children struggle with mathematics. It provides a finer grain size in the analysis of developmental steps that need to be taken and their significance in the subsequent development of mathematical competency. This precise identification of potential hurdles in the developmental process enables the design of more tailored and potentially more effective interventions.

*Innate versus emerging numerical abilities*. While other theoretical frameworks (e.g., Fuson, 1988; Dehaene, 1992; Butterworth, 2005; von Aster and Shalev, 2007) suggest that children are born with a notion of what a number is and understand that number words—once separated from the “numerical string” (Fuson, 1988)—are associated with quantities, the QNL model takes a different position. It posits that the connection between quantities and number words (as well as other symbolic representations) is something that develops gradually over a longer period of time. This is the reason why the framework is referred to as the model of quantity-to-number-word linkage. It makes the following three claims about the linkage between quantities and number words.

(1) Conceptual understanding of the linkage between quantities and numbers is the most critical prerequisite for subsequent acquisition of school mathematics—that is, here lies the *core deficit* of mathematical difficulties and developmental dyscalculia.
(2) All children are naturally born with a “deficit” in grasping discrete numerical quantities and, consequently, have to learn that number words are linked to particular quantities rather than to other characteristics of an object like color or usage. This is also true for small numbers, which are usually supposed to be apprehended with a single glance as children grow older (i.e., that lie within the subitizing range). The *quantity–to–number word linkage has to be acquired* via a developmental process; that is, this so-called deficit is not specific to children with developmental dyscalculia.
(3) While many children succeed effortlessly in acquiring this linkage, others need—and can effectively receive—explicit instruction.
These claims imply that early instruction in quantity–to–number word linkage should lead to long-term improvement in subsequent acquisition of mathematics during regular school instruction.

#### 1.2.2 Developmental steps

**Figure 1:**
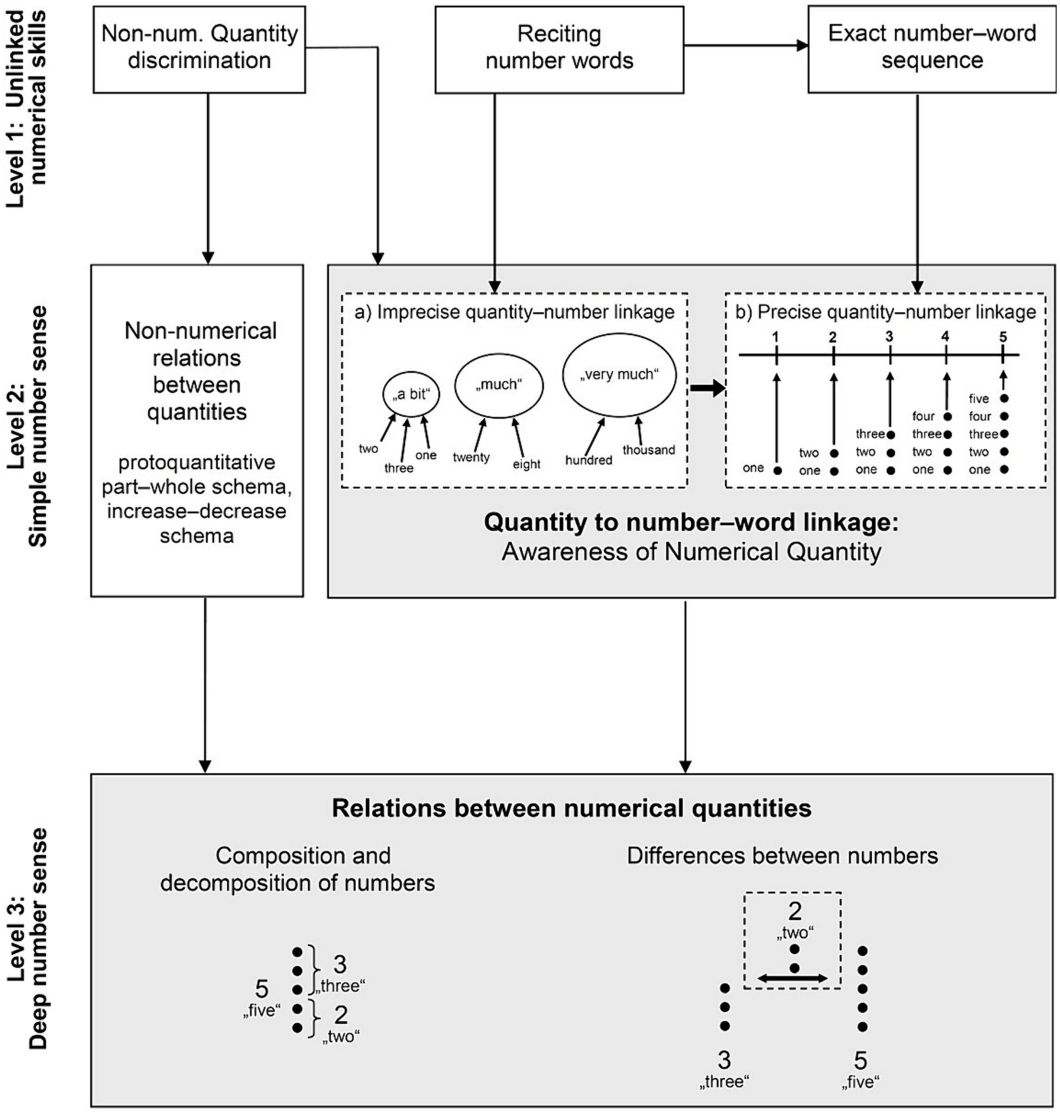
Developmental model of quantity–to–number word linkage (QNL model) (Krajewski, 2008).

In her model, Krajewski identifies three key milestones. Achieving these milestones marks and facilitates children’s progression toward a deeper understanding of the quantity–to–number word linkage (see Figure 1).

*Level 1: unlinked numerical skills (number words and numerals isolated from quantities)*. Newborns, starting at level 1 in the model, can assess whether objects or continuous quantities differ (*quantity discrimination*), effectively distinguishing between *indiscrete* amounts, i.e., between amounts that are not precisely countable but differ in scope and extent (e.g., Feigenson et al., 2002; Rousselle et al., 2004; e.g., Turati et al., 2013). Researchers have different views on whether this also implies that newborns and infants can differentiate between discrete (i.e., potentially countable) quantities at this point; however, according to the QNL model this capability is not considered necessary for normal developmental progression.

A second skill that develops in the following years but is still located at level 1 is the knowledge of *number words* and the number-word sequence. According to the QNL model, these two skills develop—at least initially—independently, that is, when children start to recite number words (e.g., “one, two, three, four, five”), they do not necessarily know that a given number word (e.g., *three*) refers to a quantity or magnitude (e.g., to all three fingers that were just counted). Rather, number words might be perceived as just reflecting a kind of labeling used when things are pointed to in sequence (e.g., *three* may be a roughly synonymous label for “middle finger”). In other words, at this early level, the use of number words by a child does not necessarily imply an understanding of their quantitative meaning.

*Level 2: simple number sense (quantity–to–number word linkage)*. Krajewski argues that children gradually learn to understand the linkage between numbers and quantities. Most children already acquire this knowledge before the beginning of formal mathematics instruction in school. In the beginning, they only have a very vague and imprecise understanding of the quantitative meaning of a number word (*imprecise quantity–to–number word linkage*, level 2a). For instance, they may understand that some number words correspond with rough verbal categories like “a bit,” that is, represent only small quantities, while other number words are associated with terms like “much” or “very much,” corresponding to large or very large quantities. For example, the number word *three* may correspond to the verbal category “a bit,” while *a hundred* might indicate “very much.” Of course, the verbal terms for magnitudes (“a bit,” “much,” “very much,” etc.) are just very rough categories that do not sharply distinguish between clearly defined number spaces. Consequently, children at this level often fail when they have to differentiate between number words that belong to the same category of verbal magnitude. Thus, if a child assigns the words *twenty-three* and *twenty-five* to the same category (e.g., “much”) he or she will not be able to tell which of these two number words represents “more” than the other. This would require a more precise categorical system than one of rough verbal categories—that is, a *precise quantity–to–number word linkage*, which characterizes the next phase of level 2 (level 2b). At this level, children are able to assign exactly countable quantities to an exact position in the number-word sequence, and therefore to differentiate even near numbers on the basis of their quantitative meaning (e.g., *twenty-four* is more than *twenty-three* and both are less than *twenty-five*), because now each number word is associated with an exactly defined quantity.

The developmental process described from level 2a to level 2b (i.e., from an initially imprecise to a precise concept of quantity) aligns with theoretical assumptions regarding the development of an approximate number sense (*ANS*) (Halberda and Feigenson, 2008). Specifically, this process involves a shift from a very imprecise representation of numbers, where the degree of imprecision increases with larger numerosities (corresponding to QNL model Level 2a), to a more precise numerical representation (corresponding to QNL model Level 2b). This transition reflects the enhancement of children’s ability to differentiate between quantities more accurately, thereby improving their overall numerical understanding and competence.

At the same time as children become able to elaborate these relations between the quantities indicated by number words, children also gather experiences with *relations between quantities (without reference to number words)*. They become aware that the size of a non-numerical quantity only changes if something is added or taken away, and that quantities can be divided into pieces that, when put together again, equal the original quantity (see also Resnick, 1989, on the *protoquantitative decrease/increase* and *part–whole schema*).

*Level 3: deep number sense (concept of number relationships)*. At the third level, the awareness of relations between non-numerical quantities (that has emerged at level 2, see above) is transferred to *numerical relations*. Children now grasp that when a discrete number of items are divided into smaller pieces, both the resulting subsets and the *relation* between these subsets can themselves be represented (exactly quantified) by means of number words. In practice this includes the ability to recognize that the number *eight* can be divided into the numbers *five* and *three (de/composition of numbers)* and that the subset *five* includes exactly two more than the subset *three (differences between numbers)*. The assumption upon which Krajewski’s model rests is that these insights basically have to be understood on a verbal level, that is, by the use of number words. The knowledge of Arabic numeral symbols (e.g., 8, 5, 3) may be helpful, but it is not a necessary precondition for this level of development. Nor is calculating on a symbolic level (e.g., 8–5 = 3) required.

### 1.3 Learning number words as a case of vocabulary acquisition

As outlined above, based on the *principle of conservative competence attribution* the QNL model suggests that merely saying a number word does not necessarily imply that a child precisely understands the meaning of this word. In our view, this phenomenon perfectly parallels the acquisition of any other word in our vocabulary. When a child uses a new word like *dog* for the first time (maybe just by parroting what it has heard another person say), it usually does not have a clear notion of what this word means. Rather, the exact meaning of the word *dog* has to be learned via an evolving process of comparing and categorizing, of testing and rejecting hypotheses, until the child has developed a sophisticated network of more or less distinctive attributes that constitute the mental representation (i.e., the meaning) of the word *dog* (e.g., Bloom, 2000). This representation might include: has legs, is barking, is not a cat, is a living being, or—somewhat later and more abstractly—is an animal, includes various different breeds. A very similar developmental process is necessary to acquire an appropriate mental representation of a number word. For example, in order to attain a deep conceptual understanding of the number word *six*, there are a lot of relevant attributes that have to be linked to this word. Examples of attributes that have to be acquired progressively are that *six*:

- is usually said between the words *five* and *seven* (level 1),
- is a kind of label that is often used when objects are pointed to in sequence (level 1),
- has something to do with quantities (level 2a),
- is not very much (level 2a),
- is less than *one hundred* (level 2a),
- is more than *five* and less than *seven* (level 2b),
- is exactly one more than *five* and one less than *seven* (level 3) – which is not the same as simply knowing that six is usually said *between* five and seven (see above)
- can be decomposed into *four* and *two* (level 3), and
- is the difference between *ten* and *four* (level 3).

Our notion of learning number words as a case of vocabulary acquisition seems particularly suited to underpin and to clarify the core assumption of the QNL model, that the linkage between number words and quantities has to be established in a developmental process.

#### 1.3.1 Practical implications

Both the principle of conservative competence attribution and the notion of number-word learning as a case of vocabulary acquisition imply the need for a much closer look than has been taken by other theoretical frameworks at how children acquire an understanding of number words as the representatives of numerical quantities. The linkage between quantities and numbers is not simply “on” or—in a few children, those with developmental dyscalculia—“off.” Rather, it has to be acquired passing through a developmental sequence. The QNL model gives a detailed theoretical description of this developmental sequence, including particular milestones that have to be mastered gradually and, thus, provides promising starting points for targeted interventions, starting points that might otherwise be neglected. According to the QNL model, dyscalculia prevention programs should strictly address this developmental sequence from level 1 to level 3, with a particular focus on establishing the linkage between number words and quantities (level 2).

The practical value of such a developmental model can be evaluated, first, by investigating the predictive power of the proposed quantity–number competencies for subsequent mathematical school achievement in typically-performing children. Second, it can be tested and determined whether children with math difficulties show deficits in the proposed developmental milestones. Third, a training program based on this model and designed to foster children’s mathematical understanding along the path laid out by these milestones is likely to effectively facilitate subsequent development and—in the long run—lead to better mathematical school achievement. In what follows, we will first conduct a theoretical discussion to shed light on points one and two above. Afterwards, we will take a look at existing studies on fostering numerical abilities in children, considering the third requirement in their light, before finally leading into our empirical study.

### 1.4 Validity of the described quantity–number competencies for numerical learning in children with and without dyscalculia

Longitudinal studies from many countries provide consistent evidence for the importance of the quantity–number competencies described above^1^ for primary mathematical school achievement when assessed before or around school entry (e.g., Passolunghi et al., 2007; Jordan et al., 2012; Watts et al., 2014). For example, Aunola et al. (2004), in a Finnish longitudinal study, found that level-1 competencies (reciting the number-word sequence forwards and backwards from a given starting point) predicted 38% of the level and 5% of the growth rate of central domains of first-and second-graders’ elementary mathematical school curriculum. Moreover, in a German longitudinal study by Krajewski and Schneider (2009a), 24% of the variance in mathematical school achievement at the end of fourth grade was predicted by level-2 competencies (quantity–to–number word linkage) that had been assessed in kindergarten 4 years before, while in turn 58% of level-2 competencies were predicted by competencies located on level 1. Similarly, Ennemoser et al. (2017) found that quantity-number competencies assessed at school entry explained 42% of the variance in math achievement by the end of 4th grade, even after controlling for nonverbal intelligence and arithmetic fact retrieval. In sum, quantity–number competencies are the strongest known predictor of later mathematical school achievement in normal-performing children. Moreover, these predictions reliably distinguish between primary school children with and without mathematical learning difficulties. Children with mathematical difficulties consistently show deficits on all three levels of quantity–number competency, in various domains: naming, reading, and writing numbers, arranging numbers on a scaled number line as well as reciting number words (all situated at level 1); matching Arabic numerals with representations of their magnitudes and comparing numbers according to their cardinal value (level 2); deficits in part-whole relations of numbers (level 3) (e.g., Gaupp et al., 2004; Geary et al., 2004; Landerl et al., 2004; Krajewski and Schneider, 2009a; Sinnakaudan and Ghazali, 2015).

Cross-sectional and longitudinal data provide important evidence for the assumptions of the QNL model. Additionally, intervention studies can make a particularly significant contribution by testing whether fostering QNL competencies, as defined by the model, indeed enhances future mathematical competence development. The evidence available to date and the research needs in this area are discussed in the following sections.

### 1.5 Evidence from early interventions to foster mathematical achievement

#### 1.5.1 Essential criteria for the evaluation of QNC-based mathematics interventions

Merely demonstrating that quantity-number competencies (QNC) substantially predict subsequent mathematical skill acquisition is not enough to fully test the theoretical assumptions of the QNL model. Given the perspective that mathematical difficulties are rooted not in a deficient number module but in a developmental delay in QNC—a delay that can be addressed through targeted intervention—it is critical to demonstrate the efficacy of such interventions. Three aspects are essential in this regard. First, it should be shown that QNC can be effectively fostered—particularly in children with weak initial skills (i.e., those with a developmental delay). Second, these effects should be sustainable, persisting beyond the intervention period. Third, it is crucial that the benefits of interventions extend beyond the trained QNC, transferring to mathematical skills that are subsequently taught during the primary school curriculum, as measured by a standardized, curriculum-based mathematics achievement test.

#### 1.5.2 The challenge of heterogeneity in mathematics intervention research

Given the abundance of research on math interventions published in the last two decades, it is surprising that the evidence specifically addressing the three aforementioned criteria remains notably incomplete. One main reason is the heterogeneity of research in the field of mathematics interventions, a diversity that is also reflected in the available reviews and meta-analyses (e.g., Mononen et al., 2014; Wang et al., 2016; Jitendra et al., 2021; Myers et al., 2022; Schnepel and Aunio, 2022; for a detailed overview, see Svane et al., 2023). Studies exhibit considerable variability, covering a range of age groups (from 2 years old to adulthood), targeting diverse populations (including unselected samples, various “at-risk” groups, and children with learning difficulties), and encompassing different educational settings (such as kindergarten and special education programs). There is also a considerable variability in the primary focus of the interventions, with some focusing narrowly on specific mathematical skills such as counting skills or arithmetic. Others take a more comprehensive approach, integrating a broader spectrum of mathematical abilities, and sometimes they even incorporate activities from other domains, such as reading (Svane et al., 2023). In the following sections, we provide an overview of studies that specifically focus on fostering QNC.

#### 1.5.3 Evidence from QNC-based interventions: short-term specific effects

Studies with a specific focus on quantity-number competencies (QNC), in line with the QNL model, are predominantly conducted in early childhood education settings and in the early years of formal schooling. Encouragingly, these studies consistently indicate that targeted interventions can effectively enhance QNC across a diverse range of groups, including children with initially weak QNC, i.e., those at a high risk of developing math difficulties (Mononen et al., 2014). However, this addresses only the first of the three criteria previously mentioned. Findings related to the two remaining aspects—the sustainability of effects and their transfer to later (curriculum-based) mathematics performance—are largely lacking. Intervention studies in the field rarely include a follow-up assessment to verify the longevity of effects over time, and even fewer studies explicitly examine whether training-induced gains in QNC actually translate into improved school mathematics performance (Mononen et al., 2014). Table 1 provides an overview of 20 intervention studies that focus on promoting foundational mathematical abilities. It details the setting (kindergarten vs. school), the characteristics of the sample (unselected children, children at risk for mathematical difficulties, children with low performance in mathematics, or those diagnosed with developmental dyscalculia), and whether a control group was included, specifying if it was an active control group (i.e., one that received an alternative intervention to control for attention effects). Additionally, the table reports the types of effects examined in each study, distinguishing whether the research focused solely on training-specific effects, on targeted skills or also explored transfer effects on non-targeted aspects of mathematical achievement. It also differentiates whether these effects were examined in the short-term or long-term. The overview, while potentially not exhaustive, effectively highlights the aforementioned challenges. For a detailed description of these studies, including what was investigated, how it relates to the QNL model, and which methodological weaknesses might have been present, please refer to Supplementary material.

**Table 1:** Interventions targeting foundational mathematical skills.

Authors/Intervention	Setting	Control group(s)	Sample	Short-term effects		Long-term effects	
				Specific^a^	Transfer^b^	Specific^a^	Transfer^b^
Griffin et al. (1995) Number Worlds	Kindergarten	Yes (active)	Unselected	Sig.	Sig.^c^	–	–
Sterner et al. (2019) Number Worlds	Kindergarten	Yes (active)	Unselected	Sig.	–	n.s.	–
Additional Early Mathematics	Kindergarten	Yes	At-risk	Sig.	–	Sig.	–
Van Luit and Schopman (2000) Additional Early Mathematics	Kindergarten	Yes	At-risk	Sig.	n.s.	Sig.	–
Wilson et al. (2006) The Number Race	Kindergarten	No	Unselected	Sig.	–	–	–
Wilson et al. (2009) The Number Race	Kindergarten	Yes	Unselected	Sig.	–	–	–
Räsänen et al. (2009) The Number Race	Kindergarten	Yes (active)	At-risk	Sig.	n.s.	–	–
Hellstrand et al. (2020) The Number Race	School	Yes	At-risk	n.s.	n.s.	–	–
Obersteiner et al. (2013) The Number Race	School	Yes (active)	Unselected	Sig.	–	–	–
Siegler and Ramani (2008) Numerical Board Game	Kindergarten	Yes (active)	Unselected	Sig.	–	–	–
Whyte and Bull (2008) Numerical Board Game	Kindergarten	Yes (active)	Unselected	Sig.	–	–	–
Hawes et al. (2019) Numerical Board Game	Kindergarten	Yes (active)	Unselected	Sig.	–	–	–
Kucian et al. (2011) Rescue Calcularis	School	No	Developmental Dyscalculia	Sig.	n.s.	–	–
Käser et al. (2013) Rescue Calcularis	School	Yes (waiting list)	Low achievers^d^	Sig.	Sig.	–	–
Fischer et al. (2011)	Kindergarten	Yes (active)	Unselected	Sig.	–	–	–
Link et al. (2013)	School	Yes (active)	Unselected	Sig.	–	–	–
Hyde et al. (2014)	School	Yes (active)	Unselected	Sig.	–	–	–
Fuchs et al. (2013) Galaxy Math Program	School	Yes	At-risk	Sig.	–	–	–
Fuchs et al. (2014)	School	Yes (active)	Unselected	Sig.	Sig.	–	–
Praet and Desoete (2014)	Kindergarten	Yes (active)	Unselected	Sig.	–	Sig.	–

–, not assessed; Sig., significant; n.s., not significant.

a Effects on training specific tasks.

b Transfer effects on non-trained numerical or arithmetic tasks.

c Transfer effects were assessed 4 weeks after training, which we did not consider indicative of long-term effects.

d Selection based on teacher judgment.

#### 1.5.4 Limited evidence for long-term and transfer effects on later mathematics performance

As illustrated in Table 1, nine of the reported studies examined the effectiveness of different mathematics intervention programs (e.g., Number Worlds, Numerical Board Game, The Number Race) in unselected samples of kindergarten children. While all of them reported short-term training effects on the targeted numerical skills, only two also investigated the sustainability of these effects, with only one providing evidence for significant long-term improvements in the trained numerical abilities.

Three additional studies also examined the effectiveness of various intervention programs in a kindergarten setting (e.g., Additional Early Mathematics), specifically focusing on children identified as being at-risk based on their low mathematical skill levels. Again, all three studies reported significant short-term training effects on the targeted numerical skills. Furthermore, two of these studies examined long-term effects and found supportive evidence for the sustainability of the interventions on numerical abilities that were specifically trained.

Eight studies investigated the effectiveness of early mathematics interventions for school children, with four using unselected samples (e.g., Fuchs et al., 2014) and four focusing on school children with mathematical difficulties (Rescue Calcularis, Galaxy Math Program). Except for the study by Hellstrand et al. (2020, an evaluation of The Number Race) all reported significant short-term effects on the trained numerical abilities. However, none of the eight school studies examined long-term effects on mathematical abilities. Moreover, none of the 20 intervention studies examined long-term transfer effects on mathematical abilities that were not targeted by the training or even included a standardized curriculum-based mathematics test, which would be particularly noteworthy.

In sum, there is a clear lack of evidence regarding long-term effects, particularly long-term transfer effects related to subsequent school achievement in mathematics. It is important to note that this conclusion is not based on a large proportion of insignificant results, which would not prove the absence of an effect (Edelsbrunner and Thurn, 2024), but on the gaping lack of studies that even explore these aspects.

### 1.6 The training of quantity–to–number-word linkage “Mengen, zählen, Zahlen” (QNL training)

#### 1.6.1 Theoretical background

Most of the training approaches mentioned above are based on theories positing the existence of an innate core deficit in number sense among children with developmental dyscalculia. The program Mengen, zählen, Zahlen (Krajewski et al., 2007) is founded on Krajewski’s model of quantity–to–number word linkage that proposes that the basis of dyscalculia and mathematical difficulties lies in a delay in children’s numerical development rather than an inherent core deficit. Consequently, the aim of the program is to close developmental gaps by tracing the developmental sequence as described in the QNL model in order to prevent mathematical difficulties or developmental dyscalculia, respectively. It is important to note that the QNL training does—of course—comprise the use of number words and corresponding numerals, but it does not contain any formal arithmetic tasks at the symbolic level.

#### 1.6.2 Structure and succession of the training

The QNL training was originally developed for five-to six-year-old kindergarten children. In its original kindergarten version, it is conducted three times per week in small groups with four to six children over the course of 8 weeks (in all, 24 sessions at 30 min each). In several studies with at-risk first graders, which are described in the following section, the program was condensed to twelve 45-min sessions conducted twice a week. The initial focus of the training is on exercising foundational numerical skills, for example, naming verbal and Arabic numerals, or comparing non-numerical quantities. During the corresponding sessions, number-word sequences and Arabic numerals up to 10 are introduced (e.g., which number belongs between two other numbers; level 1) with a focus on the awareness that individual verbal and Arabic numerals are associated with particular quantities (*quantity–to–number word linkage*, level 2; e.g., which number word belongs to a given discrete quantity). Afterwards, children are trained to understand that numbers placed later in the number-word sequence are associated with larger quantities (*number seriation*, level 2). Here children learn that ascending numbers represent an increase in discrete pieces/items or in amounts/quantities of space, while descending numbers represent fewer items or lesser amounts. The aim of the final section is to facilitate the awareness that numbers are related to each other in a particular way (*number relations*). Corresponding exercises support the understanding that numbers can be decomposed into smaller numbers and that pooling these subsets in turn leads to the original number again (de/composition of numbers, level 3; e.g., learning which number results when we put *two* and *three* together and what numbers (can) result when we break *five* apart). A further set of exercises aims at promoting children’s understanding that the difference between two numbers can be exactly described by a third number (the *difference between numbers*, level 3; e.g., finding out what number includes two more than *four*).

#### 1.6.3 Additional principles of the program: accounting for language, working memory, executive functioning

Additional principles of the program proactively consider relevant third variables that might impact the training’s effectiveness. Numerous studies have shown that deficits in mathematics are associated with deficits in other areas. These include, in particular, language competence (Purpura and Ganley, 2014; Lin et al., 2021), working memory (Friso-van den Bos et al., 2013; Zhang et al., 2022), meta cognition (Schneider and Artelt, 2010; see also Baten et al., 2017) and executive functions (Simanowski and Krajewski, 2019; Ribner et al., 2023). Although a focused mathematics training program cannot directly enhance general language competence, it can notably emphasize the use of mathematical language. To achieve this, the training extends beyond linking numbers to number words by also incorporating how to verbally express quantity-related situations. Appropriate verbal descriptions of such situations are introduced and practiced. Thus, children are guided to understand relevant principles not only visually (e.g., “Larger numbers include more space”) but also to describe and justify them using language (e.g., “Four is bigger than three because four includes more pieces than three”).

**Figure 2:**
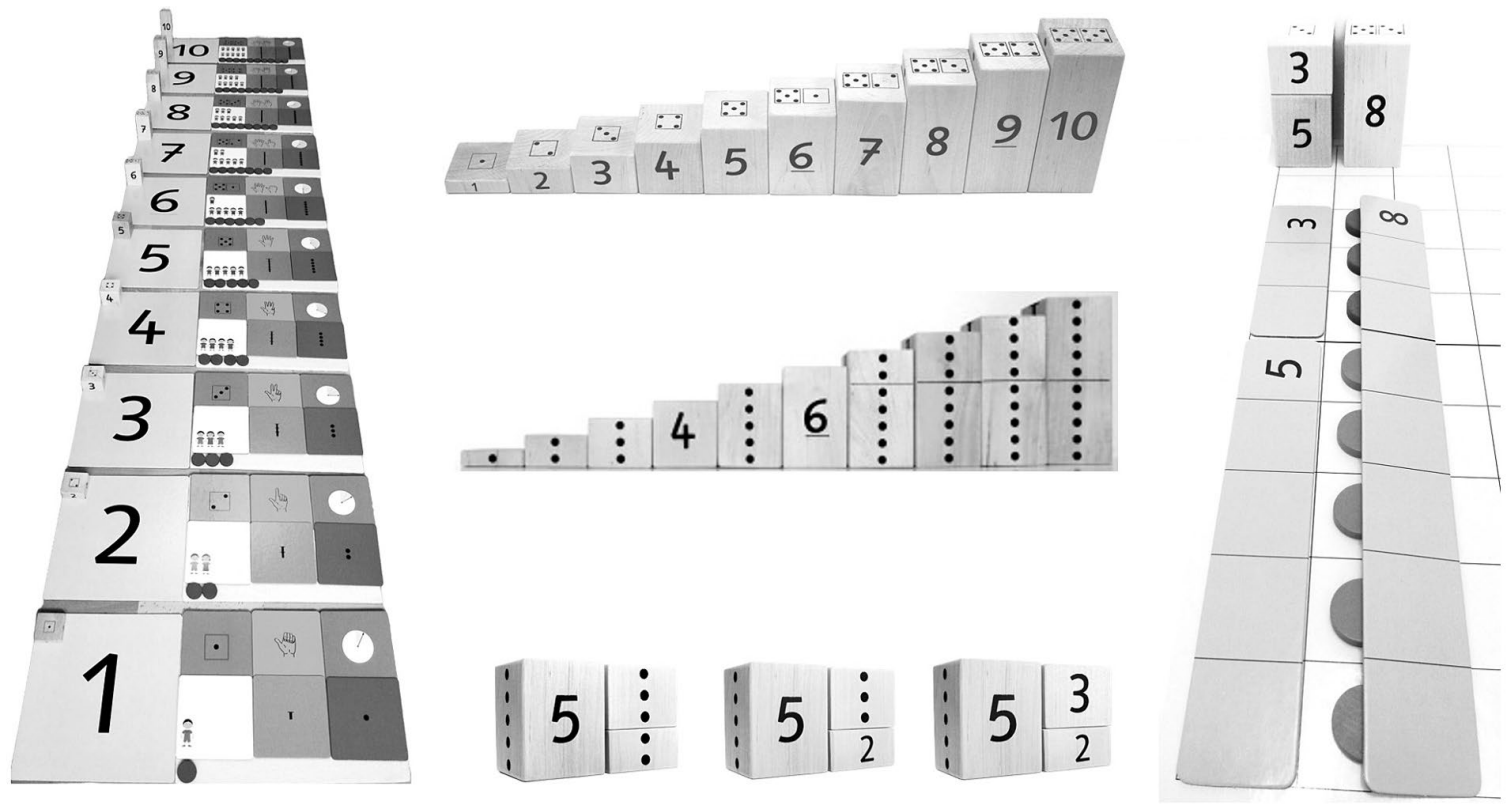
Examples of training materials from the “Mengen, zählen, Zahlen” program for teaching quantity-to-number word linkage (QNL training); “Numerical Street” on the left and “Number Staircase” on the right (Krajewski et al., 2007).

The program also takes into account low working memory and executive functions by utilizing materials that are particularly suited to facilitate the development of accurate mental representations of numbers. Initially lacking a precise internal representation of quantities, children benefit from external representations provided by the program that vividly illustrate numbers and their relationships. Thus, the QNL training puts a special emphasis on ensuring that external representations make the numerical aspect visible in isolation, i.e., a clear focus is set on quantity as a particular feature of objects (for an example, see Figure 2). Any “seductive details” or narrative contexts that might distract children’s attention from the numerical focus are avoided (e.g., Mayer et al., 2001). This approach not only alleviates the burden on working memory but also diminishes the demands on executive functions, such as discriminating and inhibiting irrelevant information.

### 1.7 Empirical evidence for the effectiveness of the QNL training

#### 1.7.1 Evidence from studies with unselected kindergarten children

In a pilot study (Krajewski et al., 2008) the first version of the QNL program by Krajewski et al. (2007), was evaluated with a sample of 260 five-to six-year-olds, assigned to four groups. One year before school entry, a first group of children was trained by kindergarten teachers using the *QNL program*, while a second group received training focused on *inductive reasoning* (Klauer, 1989) and the third group participated in another mathematical training program that was conducted as standard in their kindergarten (*Zahlenland* [Numberland]; Friedrich and de Galgóczy, 2004). In contrast to the QNL training, this program is based on the concept of introducing numbers through stories, personifying them as characters (i.e., introducing the number 2 as a swan). A fourth group served as a control group that took part in the regular kindergarten program and did not receive any specific training. An immediate posttest and a seven-month follow-up measure in kindergarten, respectively, displayed significant short-and long-term effects in quantity–number competencies for the QNL group as compared to all other groups. Further, these effects were limited to numerical performance; the four groups did not differ in their developmental growth of phonological awareness, which reflects that the QNL training specifically facilitated mathematical development. Next, at the end of the first grade (19 months after training), a second follow-up was conducted to evaluate long-term effects on mathematical school achievement: the QNL group performed better in basic arithmetic than the students who had experienced the alternative mathematical training, where numbers were introduced embedded in a narrative context. That is, there were significant long-term transfer effects of QNL training as compared to an alternative math training method. Unexpectedly, however, these effects were not confirmed in comparison to the control group.

A study conducted by Hauser et al. (2014) involving 329 kindergarten children also incorporated the QNL training. Unfortunately, the training was not carried out as outlined in the manual; for example, the recommended group size of a maximum of six kindergarten children was substantially exceeded, with up to 24 children per group. Deviations of this magnitude are very likely to compromise the intended quantity and quality of teacher-child interaction. Consequently, the study could not replicate the short-term effects of QNL training compared to another training. However, given the inadequate training fidelity, it does not allow for valid conclusions about the program’s effectiveness.

#### 1.7.2 Evidence from studies with at-risk kindergarten children

Renner et al. (2024) conducted a study to specifically evaluate the QNL program as a secondary preventive measure using a cohort of 567 preschoolers. Among them, 190 were identified as at-risk for developmental dyscalculia based on their performance in a test of quantity number competencies. The participants were divided into three distinct groups: the first group received QNL training, the second participated in the previously mentioned “Zahlenland” program (Numberland, NL) (Friedrich and de Galgóczy, 2004), and the third group followed the standard preschool curriculum. The study found that, both short-term and long-term, children in the QNL group showed significantly more improvement in quantity-number skills compared to those in the NL program and the control group (CG). Notably, 1 year after the training, at the end of 1st grade, the QNL group demonstrated enduring long-term transfer effects on arithmetic comprehension (compared to NL, *d* = 0.90 and to CG, *d* = 0.58). However, these effects did not extend to a standardized test that was aligned with the first-grade curriculum.

#### 1.7.3 Evidence from studies with children deferred from school entry

Ennemoser (2010) evaluated the effectiveness of the QNL program in a classroom setting with children at risk who had been deferred from regular school entrance. While the control group received the regular math curriculum, the training group’s first lessons of the school year were replaced by the QNL training. Results showed short-term effects for the QNL program in quantity–number competencies (QNC) compared to the control group. There were large effects on QNC level 2 (*d* = 1.29) and QNC level 3 (*d* = 0.94); these effects were already observable after only half of the program had been conducted. Transfer and long-term effects were not investigated in this study.

Hasselhorn and Linke-Hasselhorn (2013) also applied the QNL program to at-risk children who had been deferred from regular school attendance, but in this study, children were instructed in a small group setting. One group of children was trained with QNL, while the other served as a waiting control group. At posttest, the QNL group was superior to the control group in mathematical thinking, with a large effect size (*d* = 4.6). Although the subsequent training of the waiting control group constituted only a shortened version of the QNL training, large effect sizes were observed here as well (*d* = 2.5). However, this study had a very small sample size (*n* = 8), and neither transfer effects nor long-term effects were investigated. Furthermore, because no additional treatment was provided in the control condition, effects might not conclusively be attributed to the particular contents of the training.

#### 1.7.4 Evidence from studies on children with special needs or intellectual disabilities

A further school study by Sinner and Kuhl (2010) was conducted in schools for children with special needs. Forty children were trained with 12 selected sessions of the QNL program, while another group received a training of inductive reasoning (*IR*) (Klauer, 1989). Compared to the control training the QNL training had a medium effect on quantity–number competencies (*d =* 0.56). In this study, both long-term effects and transfer effects on basic arithmetic were investigated. However, both analyses yielded insignificant results, indicating that training-induced advantages were not stable over time and did not transfer to general mathematics achievement.

Similar results were found in a study by Kuhl et al. (2012) with two groups of students with intellectual disabilities. While one group received a shortened version of the QNL training (in which level-3 exercises were omitted), the other group participated in language training based on the principles of the dialogic reading approach (Whitehurst et al., 1988). Compared to the language training, the QNL training led to higher gains in the student’s quantity–number competencies (*d =* 0.44). However, this effect was only observable immediately after training, and did not persist until follow-up. Transfer effects on arithmetic were not investigated in this study.

#### 1.7.5 Evidence from studies with first grade at-risk children

The first QNL intervention study in a regular school setting was conducted by Ennemoser and Krajewski (2007). They compared two groups of first-graders with mathematical difficulties, who received either the QNL program or a reading training. The QNL training was applied in the last quarter of the first school year, which is substantially later than in the other studies reported above. A curriculum-based mathematics test was administered at pre-and posttest in order to investigate training effects. The results confirmed medium-to-large effects of the QNL training compared to the reading training. Superior achievement gains were evident in the total math curriculum test score (*d =* 0.58) and in subtests on part–whole relationships (*d =* 0.79) and word problems (*d =*0.85). These results indicate that QNL training with low-achieving first-graders at the end of first grade has short-term transfer effects on mathematical school achievement. Again, long-term effects were not investigated.

In another study, Ennemoser et al. (2015) conducted a study with 238 first-graders, of whom they identified 64 as being at risk of developing mathematical difficulties. Of these children, 32 were assigned to the training group and participated in 10 selected sessions of the QNL training. A second group of 32 children served as a control group, receiving remedial mathematical instruction provided by the school. The QNL training group outperformed the control group in the quantity–number competencies that were focused on. Both short-and long-term effects (3 months after training) were observed, with effect sizes in the medium range (*d* = 0.64 and *d* = 0.69, respectively). While the transfer effect on arithmetic was not significant immediately after training, the QNL group subsequently displayed significantly larger improvements in basic arithmetic than the control group (*d* = 0.52). This time-lagged effect indicated that the students’ progress in quantity–number competencies as a result of QNL might have continued to facilitate more efficient learning during regular mathematics instruction in school. Unfortunately, no comprehensive curriculum-based test of mathematics achievement was applied, so this interpretation of the transfer effect remains restricted to the children’s performance in basic arithmetic.

#### 1.7.6 Evidence from studies in a regular first-grade classroom setting

Finally, Olyai et al. (2014) examined the effectiveness of QNL training implemented in a regular classroom setting in school. A total of 842 first-graders were assigned to one three groups. One group received QNL training, another group underwent self-regulation (SR) training, and a third group received a combination of QNL and self-regulation training (QNL + SR). A fourth group received regular mathematics instruction and acted as the control group (CG). The QNL group displayed significantly larger short-and long-term improvements in basic arithmetic compared to the SR group and the control group. Similar gains were reported for the combined training (QNL + SR), but no evidence was found for an added value of the additional self-regulation component. The study also investigated transfer effects to a standardized, curriculum-based mathematics test and found no significant differences between the four groups on this measure.

#### 1.7.7 Summary

All prior studies that implemented the QNL training with fidelity consistently demonstrate significantly greater improvements in quantity-number competencies than the control groups. Most of these studies included active control groups that had received alternative treatments. These results constitute a growing body of evidence that QNL training can effectively stimulate the numerical development of children and is a suitable means of prevention of math difficulties. Particularly encouraging are the follow-up assessment results of several studies with low-achieving first-graders in regular school settings. These studies demonstrate that there is hope for *long-term* training effects, both in the quantity–number competencies that are actually the focus of training and, importantly, also in basic arithmetic, which can be considered a transfer effect. Nevertheless, there is currently insufficient evidence to confirm long-term transfer effects on (curriculum-based) mathematics achievement. Such confirmation is crucial, serving both as a primary criterion for evaluating the effectiveness of a prevention program and as a key validation of the QNL model’s theoretical assumptions. It would specifically substantiate the idea that mathematical difficulties are rooted in a developmental delay in QNC, which is remediable. In other words, bridging this developmental gap in QNC can significantly enhance the subsequent acquisition of more advanced mathematical skills, beyond foundational numerical competencies.

### 1.8 Aims of the study and research questions

As outlined above, quantity–number competencies provide a promising starting point for the prevention of developmental dyscalculia. However, although previous studies on various trainings report on positive results, conclusive empirical evidence for the preventive potential of numerical training is still lacking. First, a large proportion of the interventions investigated by these studies do not constitute comprehensive training programs that trace the developmental sequence in which mathematical abilities are acquired; instead, they focus on particular facets or task formats. Second, long-term effects and—most importantly—transfer effects on subsequent mathematical school achievement are scarcely addressed in the available studies. Third, many studies fail to meet basic methodological challenges (e.g., inclusion of appropriate control conditions) and do not allow reliable conclusions about the effectiveness of the particular intervention to be drawn.

Against this background, the goal of the present study was to evaluate the effectiveness of a small group prevention program for children at risk of developmental dyscalculia. Our main research question was, “Does a training program aligned with the developmental sequence of mathematical competencies, as outlined in the QNL model, effectively prevent math difficulties?” In line with the assumptions of the model, we expected that the training would yield both significant short-and long-term effects specific to the training focus (i.e., on QNC) as well as transfer effects on subsequent mathematical school achievement. Further, we addressed the question of whether transfer effects on school achievement, if observable, are mediated by preceding gains in quantity–number competencies. According to the prerequisite role of these competencies for subsequent success in the mathematical school curriculum, this mediation effect was to be expected. To address the methodological weaknesses of many prior studies, potential achievement gains from sources other than QNL training (e.g., those due to attention effects, repeated testing effects, or natural development) were controlled for by the inclusion of two appropriate control conditions.

## 2 Materials and methods

### 2.1 Participants and design of the study

A total of 575 first-grade students (mean age 6;11 years) from 14 primary schools participated in a standardized test of quantity–number competencies (MBK 1+; see below), 4 months after school entry. The assessments were conducted by trained student test administrators following a rigorously standardized procedure to ensure that the testing conditions were consistent for all children. Based on their performance in the MBK 1+, 119 of the children were identified as being at risk of math difficulties (below 20th percentile) and assigned to (quasi-)experimental conditions. Initially, a randomized assignment of participants was planned. However, during the randomization process, it became evident that diffusion effects were likely to occur, potentially undermining treatment validity (Cook and Campbell, 1979). Specifically, assigning different groups within the same school or neighboring schools, which sometimes cooperate closely, to different conditions could lead to teachers sharing information and adjusting their methods. To avoid these issues and maintain treatment validity, we opted for spatial assignment instead of randomization, ensuring that groups assigned to different conditions were not from the same or closely collaborating schools. Half of the at-risk sample (the QNL group, *n* = 61; 34 girls and 27 boys) received the training in quantity–to-number-word linkage, as described above. The other half was assigned to the two control groups, one of which (*n* = 30; 15 girls and 15 boys) participated in training in inductive reasoning (Klauer, 1989; see also Klauer and Phye, 2008; this was the IR group), while the remaining group did not receive any additional training (the control group, CG, *n* = 28; 16 girls and 12 boys). All trainings were conducted in a small-group setting with 3 to 7 children.

**Figure 3:**
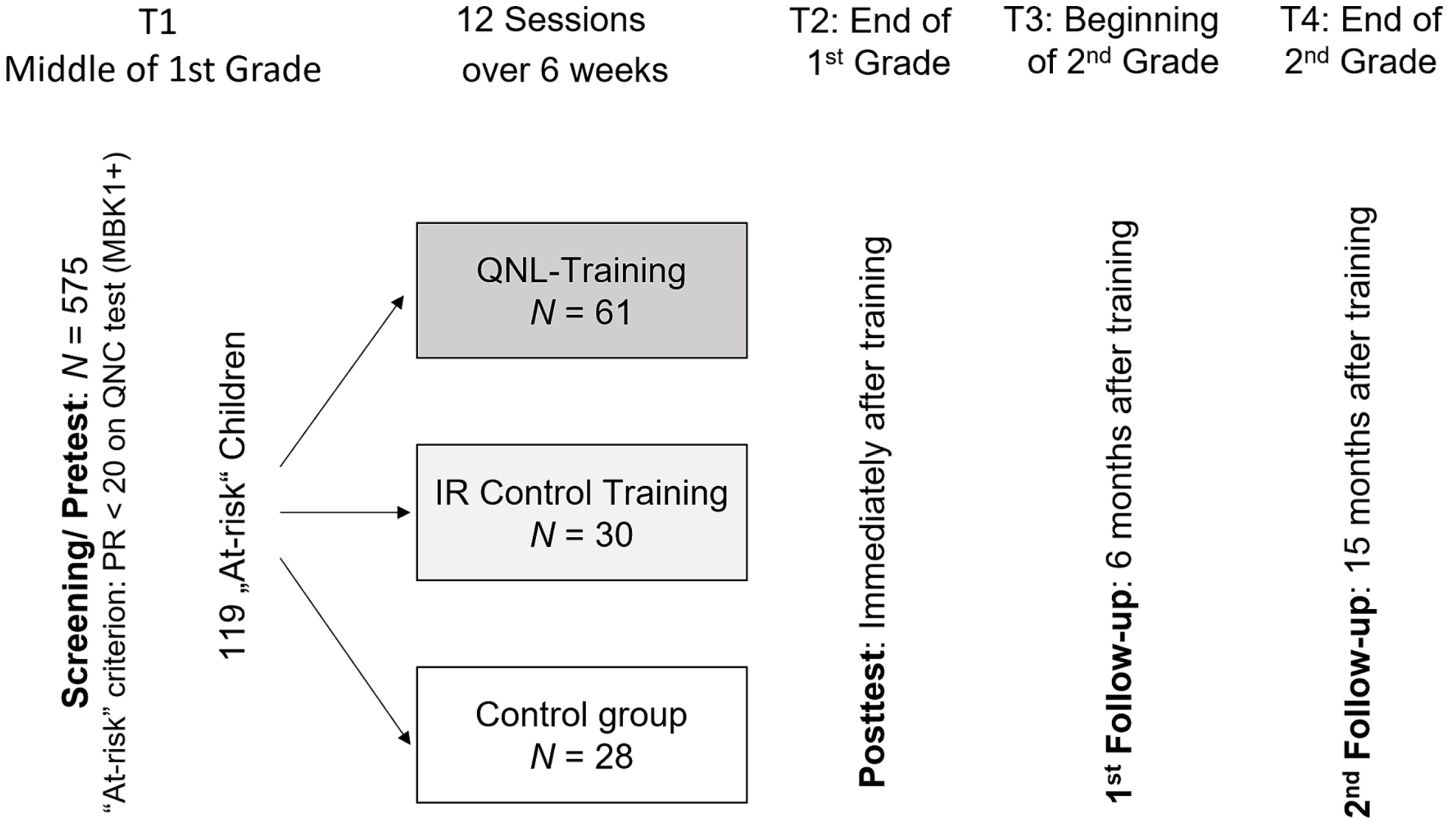
Design of the study.

As shown in Figure 3, four measurement points were set: in addition to the pre-and posttest (T1, T2), two follow-up sessions were included in order to assess long-term effects. These two additional measurement points were, respectively, 6 months (T3) and 15 months after training (T4; end of second grade). Quantity–number competencies were measured from T1 until T3; mathematical achievement was assessed at all measurement points (T1 until T4).

Until T4 (i.e., 15 months after training) eight children had moved to another town or school, respectively, and further four children did not participate in the final follow-up session because of illness. This constitutes an attrition rate of 10%. Six of these children dropped out of the QNL group, while each of the other two groups lost three participants.

### 2.2 Measures

#### 2.2.1 Quantity–number competencies at T1, T2, and T3

As a measure of quantity–number competencies (as described under the QNL model), we used a standardized test (*Test mathematischer Basiskompetenzen in der ersten Klasse; MBK 1+*; Ennemoser et al., 2017), containing the following subtests. To tap *QNC level 1*, first, 10 number words were presented orally to the children, who had to write down the corresponding Arabic numerals (*number-word transcoding*). Next, children were given pairs of numerals (e.g., 11 and 13) and had to fill in the number missing between them (12) (number gaps). Four subtests were given to assess mastery of *level 2*. In these subtests children were required to link quantities to numbers (*numerical quantities*), to compare the numerical size of numbers (*number comparison*), to place numbers on number lines (*number lines*), and to fill in gaps in several series of numerical quantities (*number seriation*). Finally, in order to assess competencies on *level 3*, children were asked to determine which number is exactly one more (or one less, respectively) than a given number (*one more, one less*), to complete part–whole tasks where either one or two numbers were decomposed into two components (*decomposition, part–whole*) and, finally, to solve word problems presented as comparison problems (see Riley et al., 1983). Each correct solution was given 1 point, except for number-word transcoding (0.5 points). The maximum score on the QNC test was 49. Reliability was 0.90 (Cronbach’s α).

#### 2.2.2 Mathematical school achievement at T1, T2, T3, and T4

Since no standardized test for mathematical school achievement was available for the initial months of the school year, we utilized the supplemental *basic arithmetic test* included in the MBK 1+ (see above) to evaluate the children’s basic arithmetic at T1. In 8 s, children had to solve as many addition tasks as possible, up to 10 (maximum score = 20, Cronbach’s α = 0.92). At the end of first grade (T2) and the start of second grade (T3) the standardized German mathematics test *Deutscher Mathematiktest für erste Klassen* (*DEMAT 1+*; Krajewski et al., 2002), which is based on the primary school curriculum of all German federal states, was administered to the children (maximum score = 36, Cronbach’s α = 0.82). Mathematical school performance at the end of second grade (T4) was assessed using the standardized *Heidelberger Rechentest für erste bis vierte Klassen* (*HRT 1–4*; Haffner et al., 2005). As per the test-developers, reliability is 0.93.

#### 2.2.3 Nonverbal intelligence (IQ)

In order to control for effects of intellectual ability, the short version of the *Culture Fair Intelligence Test* (*CFT 1, Scale 1*; Cattell et al., 1997) was included as a pretest measure. The short version consists of three subtests, on classifications, similarities, and matrices, respectively, (maximum score = 36; Cronbach’s α = 0.90).

### 2.3 Interventions

#### 2.3.1 Training of quantity-to-number-word linkage

The QNL training program *Mengen, zählen, Zahlen* (Krajewski et al., 2007) was used to foster *numerical development* in the first training group. The training was originally designed for kindergartners and, thus, had to be tailored for a school setting. The original program’s 24 sessions, each lasting 30 min, were condensed to twelve 45-min sessions. This was achieved by omitting five sessions focused on the introduction of the numbers one to ten and by reducing repetition exercises. The remaining sessions especially focused on number seriation and number relations (level-2 and level-3 competencies; for a detailed description see above). As a supplement to the kindergarten version of the training, additional worksheets were introduced in order to facilitate transfer of the trained level-2 and level-3 competencies to pictorial and symbolic task formats. The adapted version of the QNL training was administered over the course of 6 weeks and was conducted in groups of three to seven children. In order to strengthen treatment fidelity trainers received a four-hour training and were provided with a highly standardized manual that specifies every single session in detail (aims, materials, and relevant verbal instructions). All learning materials necessary for the training were provided as well. The delivery of sessions was recorded by the teachers. According to these records all 12 sessions were held as specified in the manual. For organizational reasons, however, in some of the groups more than 6 weeks (up to 11) were necessary to deliver the 12 training sessions.

#### 2.3.2 Inductive reasoning training

A second group of children received a training in *inductive reasoning* (*IR*; *Denktraining für Kinder 1;* Klauer, 1989). Children are encouraged to apply systematic comparison in order to identify differences and similarities (with respect to attributes and relations, respectively) and consequently to induce regularities. Inductive reasoning is hypothesized to play a crucial role in problem-solving, and there is supportive evidence that IR training has significant and long-lasting effects on non-verbal IQ and academic learning (e.g., Klauer and Phye, 2008). The training used here consisted of 120 visually presented tasks conducted in groups of five to seven children over the course of 12 sessions held parallel to those in the QNL training.

#### 2.3.3 Control group

The third group only participated in regular mathematics instruction provided by school and did not receive additional treatment during the six-week period of intervention.

### 2.4 Missing data

The analysis of missing data revealed that the percentages of missing values were low, with values across the first three measurement points ranging from 0.8% (IQ at T1) to 6.7% (math achievement at T3). The highest percentage of missing data occurred for math achievement at T4, with 10.1% missing due to cumulative relocations over the course of the study and student absences caused by illness at the last measurement point. To determine if the missing data were missing completely at random (MCAR), Little’s MCAR test was conducted. Results indicate that it is reasonable to assume that the values are completely missing at random (χ^2^ = 52.404, *df* = 46, *p* = 0.24). Multiple imputation was employed to address the missing data, resulting in 5 imputed datasets. The final analyses were conducted on the pooled imputed datasets.

### 2.5 Statistical analysis

The analyses were conducted using SPSS Version 29.0 (IBM Corp, 2023a) and AMOS Version 29.0 (IBM Corp, 2023b). Descriptive statistics for the variables were calculated for all data, and pretest differences were examined using analyses of variance (ANOVAs).

To account for the clustered data structure, with children trained in small groups, we employed multilevel modeling (MLM). Traditional linear regression assumes that all observations are independent, but this assumption is violated in our study due to the group-based training. For example, children within the same small group might perform similarly because they share the same instructor and training environment. Ignoring this grouping could lead to underestimated standard errors and biased effect sizes (Hox, 2010).

#### 2.5.1 Model specification

As previously mentioned in chapter 2.1, all at-risk students were organized into small training groups of three to seven children, which were assigned to either the QNL intervention, the IR training, or the CG. A two-level nested data structure was assumed, with students as level-1 units (*n* = 119) and training groups as level-2 units (*n* = 22).

Separate multilevel analyses were conducted to examine training effects on different outcome measures: effects on the trained quantity-number competencies (QNC) immediately after training (short-term effects) and 6 months after training (long-term effects), as well as transfer effects on mathematical school achievement at three time points: immediately after training (T2), 6 months after training (T3), and 15 months after training (T4).

Pretest QNC scores and IQ were included as covariates to control for initial levels of QNC and differences in intellectual ability when analyzing the effects on the trained competencies. For transfer effects on mathematical achievement, IQ and children’s early mathematics performance (basic arithmetic) at T1 served as covariates to control for differences in intellectual ability and initial achievement level.

We attempted to fit four models with progressively increasing complexity for each outcome variable using maximum likelihood estimation. However, the fourth model, which included random slopes, did not converge (see below). Therefore, we compared the fit of the first three models using log-likelihood ratio tests and report the results of the most appropriate model. The models were specified as follows:

##### 2.5.1.1 Model 1: null model (unconditional model)

This model did not include any explanatory variables and only the random intercept to partition the variance between groups. It served as a baseline to estimate and compare subsequent models. The intraclass correlation coefficient (ICC) was calculated to determine the proportion of variance in the outcome variable attributable to differences between groups (level-2 units).

An ICC greater than 0.10 is a commonly used as a threshold indicating that a significant portion of the variance in the outcome variable is due to differences between groups (Snijders and Bosker, 2012), which suggests that MLM is appropriate.

##### 2.5.1.2 Model 2: fixed effects model

This model included the above-mentioned explanatory variables (covariates and quasi-experimental condition) as fixed effects to assess their impact on the respective outcome variable.

##### 2.5.1.3 Model 3: random intercept model

This model included explanatory variables as fixed effects and additionally allowed the intercept to vary across different groups, acknowledging that each group might have a different baseline regarding the outcome variable.

##### 2.5.1.4 Model 4: random slopes model

This model allowed both the intercept and slopes (coefficients of predictor variables) to vary across groups, capturing potential differences in how predictor variables influence the outcome within different groups. However, as mentioned above, models with random slopes did not converge. Several attempts to resolve the convergence issues were made, including adjusting initial values and modifying convergence criteria, scaling predictor variables and simplifying the model, using other statistical software programs like R Version 4.40 (R Core Team, 2024) and Mplus Version 8.11 (Muthén and Muthén, 2024),^2^ but the problems persisted. Therefore, the estimates of random slopes models are not reported for any outcome variable. Instead, we report the results from models with random intercepts, which adequately capture the nested structure of the data and control for potential intra-group correlation.

## 3 Results

### 3.1 Descriptive statistics and preliminary analysis

Descriptive statistics for all variables and measurement points are shown in Table 2. Preliminary analyses of variance (ANOVAs) revealed no significant differences in IQ or quantity–number competencies (QNC) between the four groups at the outset of the study (*p*s > 0.05). However, there was a significant effect of group with regard to basic arithmetic [*F*(2, 113) = 7.38; *p* < 0.01]. *Post-hoc* tests confirmed that this effect was due to the IR group, which showed superior basic arithmetic compared to the two other groups (*p* < 0.05).

**Table 2:** Descriptive statistics for all variables and all measurement points (T1–T4).

			Condition			
	QNL training (QNL)		Inductive reasoning (IR)		Control group (CG)	
	*M*	*SD*	*M*	*SD*	*M*	*SD*
Quantity-number competencies (QNC)						
T1: *MBK 1+*	23.91	6.28	24.27	5.53	24.63	5.07
T2: *MBK 1+*	37.90	6.30	28.60	6.89	30.42	7.22
T3: *MBK 1+*	42.18	5.96	35.79	6.66	33.315	5.72
Mathematics achievement						
T1: *Basic arithmetic*	10.28	4.68	14.73	5.66	11.61	4.41
T2: *DEMAT 1+*	20.77	6.72	20.10	6.94	20.41	5.89
T3: *DEMAT 1*+	25.68	6.35	20.42	7.08	20.61	5.85
T4: *HRT 1–4*	45.69	4.84	44.34	6.25	42.50	4.34
Intelligence						
T1: *CFT 1*	18.42	5.43	19.60	4.74	17.82	4.14

^*^p < 0.05. ^**^p < 0.01.

The correlations between pretest, posttest, and follow-up performances in QNC and curricular mathematics performances (DEMAT, HRT) range from *r* = 0.29 to *r* = 0.62 (see Table 3). These values are within the expected range for selected at-risk samples, likely being somewhat reduced due to the limited variance in this group’s abilities and the training provided between assessments. As expected, the correlations of QNC with basic arithmetic are lower and not always significant.

**Table 3:** Correlations among pretest, posttest, and follow-up scores for the at-risk children.

			QNC			Basic arithmetic			Math achievement		
			T1	T2	T3	T1	T2	T3	T2	T3	T4
QNC											
MBK1+	T1		–								
MBK1+	T2		0.40^**^	–							
MBK1+	T3		0.32^**^	0.56^**^	–						
Basic arithmetic											
	T1		0.22^*^	0.14	0.11	–					
	T2		0.29^**^	0.28^**^	0.34^**^	0.37^**^	–				
	T3		0.15	0.21^*^	0.26^**^	0.35^**^	0.42^**^	–			
Math achievement											
DEMAT 1+	T2		0.29^**^	0.40^**^	0.36^**^	0.34^**^	0.43^**^	0.37^**^	–		
DEMAT 1+	T3		0.49^**^	0.55^**^	0.62^**^	0.16	0.48^**^	0.36^**^	0.53^**^	–	
HRT 1–4	T4		0.34^**^	0.44^**^	0.54^**^	0.41^**^	0.49^**^	0.54^**^	0.45^**^	0.69^**^	–
IQ (CFT)	T1		0.33^**^	0.39^**^	0.38^**^	0.31^**^	0.16	0.17	0.29^**^	0.34^**^	0.37^*^

^*^p < 0.05. ^**^p < 0.01.

### 3.2 Specific training effects on quantity–number competencies

The results of the MLM analyses for specific short-and long-term training effects on quantity–number competencies are summarized in Table 4.

**Table 4:** Multilevel analysis on specific training effects on QNC.

	QNC short-term effects			QNC long-term effects		
	Null model	Fixed effects model	Random intercept model	Null model	Fixed effects model	Random intercept model
Intercept	33.80** (1.14) [31.56, 36.04]	18.61** (2.39) [13.93, 23.28]	18.36** (2.42)[13.61, 23.11]	37.86** (1.07) [35.76, 39.96]	27.21** (2.49) [22.33, 32.08]	27.17** (2.52)[22.23, 32.11]
QNL training vs. control group		7.49** (1.19) [5.16, 9.82]	7.54** (1.43) [4.75, 10.34]		8.75** (1.26) [6.28, 11.21]	8.74** (1.36) [6.07, 11.40]
		*d* = 0.82	*d* = 0.69		*d* = 0.90	*d* = 0.83
QNL training vs. IR training		10.04** (1.17)[7.76, 12.33]	9.95** (1.44) [7.14, 12.76]		6.60** (1.22) [4.21, 8.99]	6.57** (1.34) [3.94, 9.20]
		*d* = 1.12	*d* = 0.90		*d* = 0.69	*d* = 0.63
Pretest-score		0.43** (0.09) [0.26, 0.60]	0.45** (0.09) [0.28, 0.63]		0.22* (0.10) [0.03, 0.41]	0.22* (0.10) [0.03, 0.41]
IQ (CFT)		0.49** (0.10) [0.29, 0.69]	0.48** (0.10) [0.28, 0.68]		0.51** (0.11) [0.29, 0.72]	0.51** (0.11) [0.30, 0.72]
*df*	3	6	7	3	6	7
Pseudo *R*^2^ (marginal)	0	0.56 (0.00)	0.56 (0.00)	0	0.46 (0.01)	0.46 (0.01)
Pseudo *R*^2^ (adjusted)	0.34	0.56 (0.00)	0.61 (0.00)	0.32	0.46 (0.01)	0.48 (0.02)
−2 Log-likelihood	806.02	729.88 (0.17)	728.13 (0.09)	793.57 (0.41)	738.49 (1.22)	737.90 (1.34)
BIC	820.36	758.56 (0.17)	761.58 (0.10)	807.91 (0.41)	767.88 (1.39)	771.35 (1.34)
ICC	0.34			0.32 (0.01)		

Values in parentheses are standard errors; values in brackets are 95% confidence intervals; BIC, Bayesian information criterion; ICC, Intra-class correlation coefficient; *p < 0.05. **p < 0.01.

#### 3.2.1 Short-term effects

Likelihood-Ratio tests indicated that both the fixed effects model and the random intercept model provided a significantly better fit than the null model (*p*s < 0.001) for the short-term effects at T2. The ICC for the null model was 0.34, indicating that the use of MLM was appropriate.

However, the random intercept model did not significantly improve the fit over the fixed effects model (χ^2^ = 1.75, *df* = 1, *p* = 0.185). Therefore, the parameter estimates of the fixed effects model are reported.

The results of the MLM revealed significant effects for the QNC pretest score (*b* = 0.43, *SE* = 0.09, 95% CI [0.26, 0.60], *p* < 0.001) and for IQ (*b* = 0.49, *SE* = 0.10, 95% CI [0.29, 0.69], *p* < 0.001). Furthermore, the QNL training group yielded significantly higher gains in quantity–number competencies from pretest to posttest than the control group (*b* = 7.49, *SE* = 1.19, 95% CI [5.16, 9.82], *p* < 0.001) and the inductive reasoning group (*b* = 10.04, *SE* = 1.17, 95% CI [7.76, 12.33], *p* < 0.001). Large effect sizes were observed, amounting to more than one standard deviation in favor of the QNL training (QNL vs. CG: *d* = 0.82, 95% CI [0.61, 1.02]; QNL vs. IR group: *d* = 1.12, 95% CI [0.89, 1.35]).

#### 3.2.2 Long-term effects 6 months after training

For QNC-specific long-term effects, the parameter estimates of the fixed effects model are reported, since the model fit significantly surpassed the null model (χ^2^ = 55.08, *df* = 3, *p* < 0.001), and the addition of a random intercept did not significantly improve the model fit (χ^2^ = 0.59, *df* = 1, *p* = 0.443).

The analysis indicated that the observed advantage of the QNL training group was persistent over time. Six months after training (T3), the QNL group still significantly outperformed both the inductive reasoning group (*b* = 6.60, *SE* = 1.22, 95% CI [6.07, 11.40], *p* < 0.001) and the control group (*b* = 8.75, SE = 1.26, 95% CI [6.28, 11.21], *p* < 0.001). Effect sizes were in a medium to large range, between *d* = 0.69, 95% CI [0.43, 0.95] (QNL vs. IR) and *d* = 0.90, 95% CI [0.63, 1.16] (QNL vs. CG).

### 3.3 Transfer effects on mathematical school achievement

The results of the MLM analyses for short-and long-term transfer effects on mathematical school achievement are summarized in Table 5.

**Table 5:** Multilevel analysis on transfer effects on mathematics achievement in school.

	Math achievement T2 (DEMAT 1+)			Math achievement T3 (DEMAT 1+)			Math achievement T4 (HRT1-4)		
	Null model	Fixed effects model	Random intercept model	Null model	Fixed effects model	Random intercept model	Null model	Fixed effects model	Random intercept model
Intercept	20.29** (0.67) [18.97, 21.61]	9.91** (2.19) [5.62, 14.20]	9.90** (2.20) [5.59, 14.21]	23.04** (0.98) [21.13, 24.96]	15.10**(2.20) [10.78, 19.41]	15.63**(2.26) [11.20, 20.06]	44.45** (0.61) [43.26, 45.64]	35.18** (1.67) [31.89, 38.46]	35.12** (1.71) [31.77, 38.47]
QNL vs. control group		0.52 (1.33) [2.10, 3.13]	0.52 (1.37) [2.16, 3.20]		4.74** (1.39) [2.02, 7.46]	4.65* (1.80) [1.12, 8.17]		2.74** (1.0) [0.79, 4.70]	2.67* (1.18) [0.36, 4.98]
		*d* = 0.05	*d* = 0.05		*d* = 0.43	*d* = 0.33		*d* = 0.36	*d* = 0.29
QNL vs. IR training		2.54 (1.45) [0.30, 5.39]	2.54 (1.48) [0.37, 5.45]		6.11** (1.55) [3.05, 9.18]	5.86** (1.98) [1.95, 9.77]		2.77* (1.15) [0.50, 5.05]	2.69* (1.32) [0.08, 5.30]
		*d* = 0.24	*d* = 0.23		*d* = 0.54	d = 0.39		*d* = 0.32	*d* = 0.27
Basic arithmetic		0.51** (0.12) [0.27, 0.74]	0.50** (0.12) [0.26, 0.74]		0.19 (0.12) [0.04, 0.43]	0.19 (0.12) [0.05, 0.42]		0.41** (0.09) [0.24, 0.58]	0.42** (0.09) [0.25, 0.59]
IQ (CFT)		0.28* (0.11) [0.06, 0.50]	0.28* (0.11) [0.06, 0.51]		0.45** (0.11) [0.23, 0.67]	0.42** (0.11) [0.21, 0.64]		0.31** (0.09) [0.14, 0.48]	0.31** (0.09) [0.14, 0.48]
*df*	3	6	7	3	6	7	3	6	7
Pseudo *R*^2^ (marginal)	0	0.23 (0.02)	0.23 (0.02)	0	0.28 (0.04)	0.26 (0.04)	0	0.33 (0.01)	0.34 (0.01)
Pseudo *R*^2^ (adjusted)	0.05 (0.01)	0.23 (0.02)	0.24 (0.01)	0.30 (0.02)	0.28 (0.04)	0.39 (0.05)	0.11 (0.02)	0.33 (0.01)	0.40 (0.01)
−2 Log-Likelihood	785.96 (1.18)	755.39 (2.40)	755.34 (2.38)	780.70 (1.88)	757.81 (2.16)	753.46 (2.49)	728.39 (2.26)	684.67 (2.68)	681.49 (2.72)
BIC	800.30 (1.19)	784.06 (2.41)	788.79 (2.38)	794.23 (1.88)	786.01 (2.17)	786.91 (2.50)	743.53 (2.26)	712.94 (2.46)	715.35 (2.49)
ICC	0.05 (0.01)			0.30 (0.02)			0.11 (0.02)		

Values in parentheses are standard errors; values in brackets are 95% confidence intervals; BIC, Bayesian information criterion; ICC, Intra-class correlation coefficient. *p < 0.05. **p < 0.01.

#### 3.3.1 Short-term transfer effects immediately after training

Since the fixed effects model again provided a better fit than the null model (χ^2^ = 30.57, *df* = 3, *p* < 0.001) and the addition of a random intercept did not significantly improve the fit (χ^2^ = 0.05, *df* = 1, *p* = 0.823), the estimated parameters of the fixed effects model are reported for short-term transfer effects at T2.

The analysis of short-term effects on mathematical school achievement, assessed by the *DEMAT 1+* immediately after training, did not parallel the findings for specific effects on quantity–number competencies. Significant effects were found only for the two covariates (basic arithmetic: *b* = 0.51, *SE* = 0.12, 95% CI [0.27, 0.74], *p* < 0.001; IQ: *b* = 0.28, *SE* = 0.11, 95% CI [0.06, 0.50], *p* = 0.014), whereas the QNL group did not significantly differ from the other two groups (QNL vs. CG: *b* = 0.52, *SE* = 1.33, 95% CI [2.10, 3.13], *p* = 0.698; QNL vs. IR: *b* = 2.54, *SE* = 1.45, 95% [0.30, 5.39], *p* = 0.08). This result parallels previous findings indicating that transfer effects may require some time and is consistent with the expectation that, immediately after training (T2), children in the QNL group may not yet be able to transfer their improved quantity–number competencies to typical task formats used in regular mathematics instruction (Ennemoser et al., 2015).

#### 3.3.2 Transfer effects 6 months after training

The results of the likelihood-ratio test indicated that both the fixed effects model and the random intercept model significantly improved the fit over the null model (*p*s < 0.001). Furthermore, the random intercept model also significantly improved the fit over the fixed effects model (χ2 = 4.35, *df* = 1, *p* = 0.037). Consequently, the estimated parameters of the random intercept model are reported.

Six months after training (T3), the effect of IQ was still significant (*b* = 0.42, *SE* = 0.11, 95% CI [0.21, 0.64], *p* < 0.001), while the effect for basic arithmetic was not (*b =* 0.19, *SE* = 0.12, 95% CI [0.05, 0.42], *p* = 0.101). However, in contrast to the result of the analysis of short-term effects, the QNL group showed superior mathematics performance to both the inductive reasoning group (*b* = 5.86, *SE* = 1.98, 95% CI [1.95, 9.77], *p* < 0.001, *d* = 0.39, 95% CI [0.14, 0.65]) and the control group (*b* = 4.65, *SE* = 1.80, 95% CI [1.12, 8.17], *p* < 0.001, *d* = 0.33, 95% CI [0.08, 0.59]), which indicates a significant time-lagged transfer effect of QNL training on children’s mathematical achievement as compared to the two control groups.

#### 3.3.3 Transfer effects 15 months after training

For long-term transfer effects, again both the fixed effects model and the random intercept model provided a significantly better fit than the null model (*p*s < 0.001). However, the random intercept model did not further improve the fit over the fixed effects model (χ^2^ = 3.18, *df* = 1, *p* = 0.074). Therefore, the estimates of the fixed effects model are reported.

Fifteen months after training (T4), significant effects were observed for the two covariates (basic arithmetic: *b* = 0.41, *SE* = 0.09, 95% CI [0.24, 0.58], *p* < 0.001; IQ: *b* = 0.31, *SE* = 0.09, 95% CI [0.14, 0.48], *p* < 0.001). Children who participated in the QNL training still significantly outperformed the two other groups 15 months after training (QNL vs. CG: *b* = 2.74, *SE* = 1.00, 95% CI [0.79, 4.70], *p* = 0.006; QNL vs. IR: *b* = 2.77, *SE* = 1.15, 95% CI [0.50, 5.05], *p* = 0.017). Effect sizes were in a small to medium range (IR training: *d* = 0.32 [0.06, 0.58]; control group: *d =* 0.36 [0.10, 0.61]).

### 3.4 Mediation model

**Figure 4:**
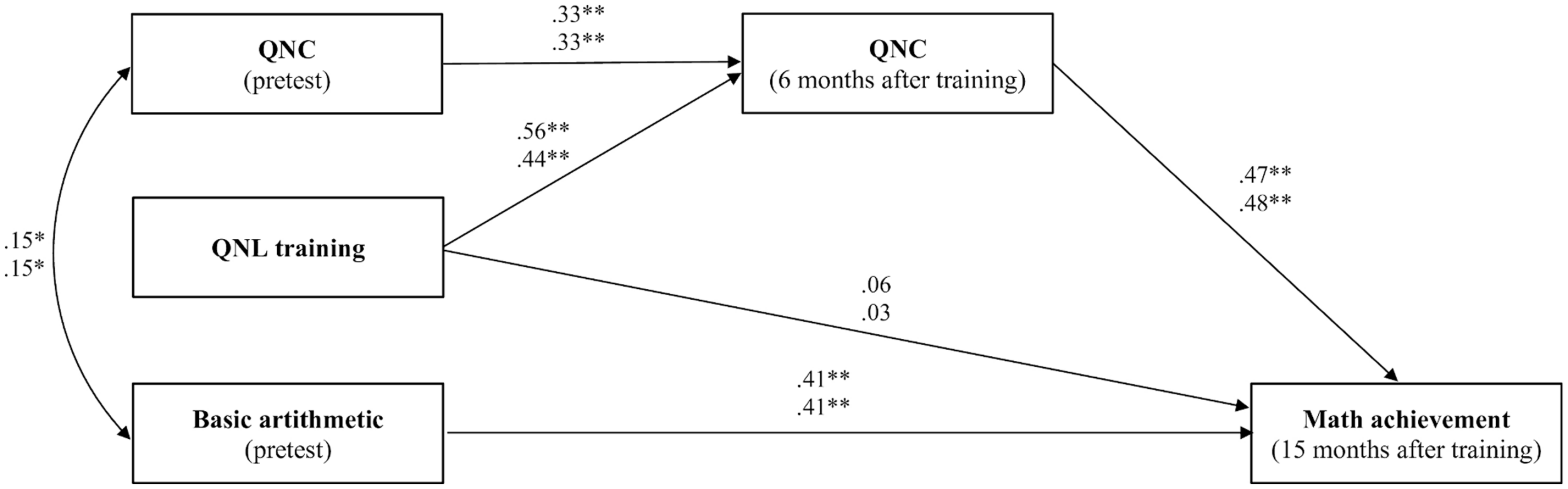
Mediation model: transfer effects of QNL training on mathematics achievement 15 months after intervention, mediated by previous (training-induced) gains in (upper values: QNL vs. control group; lower values: QNL vs. inductive reasoning group).

A path model was specified to test the hypothesis that transfer effects on mathematical school achievement are mediated by preceding (specific) effects on the development of quantity–number competencies (see Figure 4). The dependent variable was mathematical achievement at the end of the second grade. To control for differences in initial performance, basic arithmetic at the first measurement point (T1) was included as a covariate. Quantity–number competencies (QNC) at posttest were included as the mediator, adjusted for the initial QNC levels at T1, thus representing the change in QNC during the intervention phase.

Although we expected the mediation effect to hold for both control groups, we analyzed the model separately for each control group to ensure we did not miss potential differences: one model comparing the QNL training group with the control group, and another model comparing the QNL training group with the reasoning group. As can be seen in Figure 4, the relevant coefficients for the two models are presented one below the other for easy comparison (QNL vs. control group at the top, QNL vs. inductive reasoning group at the bottom). The results were closely parallel, indicating that the effects were consistent across both comparisons.

First of all, both analyses confirm that there are significant effects on QNC in favor of the QNL group (Model 1: β = 0.56; *p* < 0.001, Model 2: β = 0.44; *p* < 0.001). These findings are consistent with the training effects observed in the multilevel analyses above, indicating that the results are robust across different analytical approaches. Results further support the assumption of an *indirect training effect* on mathematical school achievement. Higher gains in QNC during the intervention phase are subsequently related to better mathematical school achievement 15 months after training (Model 1: β = 0.47; *p* < 0.001, Model 2: β = 0.48; *p* < 0.001). Controlling for this indirect (mediation) effect, the direct effect of QNL training on mathematical achievement was not significant in either model (Model 1: β = 0.06; *p*, Model 2: β = 0.03; *p* = 0.73).^3^ Both models explained similar proportions of the variance in mathematics achievement at the end of second grade (Model 1: 42%, Model 2: 43%).

## 4 Discussion

The model of quantity–to–number word linkage (i.e., the QNL model) (Krajewski, 2008) traces the development of early numerical abilities. It identifies important milestones that have to be met over the course of development and, thus, indicates promising starting points for prevention. A large body of evidence demonstrates the predictive value of the competencies described by the QNL model. With regard to its preventive potential, however, the evidence available to date is promising but not conclusive. Most findings are limited to short-term effects on specifically trained skills, while particularly long-term transfer effects on subsequent mathematical school achievement are scarcely investigated. Moreover, the available studies usually did not include a comprehensive training program specifically designed to establish conceptual understanding in a step-by-step process in accordance with the acquisition of foundational skills hypothesized by developmental theory. Given this situation, the goal of our study was to investigate the effectiveness of a strictly theory-based prevention program (QNL program) for first-grade children who are at risk of developing mathematical difficulties. We further aimed to investigate not only short-term but also long-term effects, 6 and 15 months after training. Both specific and transfer effects on subsequent mathematical school achievement were investigated; if both specific and transfer effects were observable, we intended also to test whether transfer effects on school achievement could be attributed to preceding (training-induced) gains in quantity–number competencies (that is, a mediation effect).

Our results align with and expand upon existing findings on the *effectiveness of QNL training*. Both short-and medium-term effects on quantity–number competencies were found up to 6 months later. These specific effects on foundational numerical insights, which were particularly addressed by the training, subsequently led to a long-term transfer effect on mathematical school achievement. Interestingly, this transfer effect was not yet evident immediately after training but grew significant 6 months later and remained stable over the course of the next 15 months. This time-lagged effect replicates the findings by Ennemoser et al. (2015) and is in accordance with the theoretical assumptions of the QNL model. It indicates that while the training successfully closed developmental gaps in quantity–number competencies, this increase did not immediately boost children’s performance in a standardized mathematics test at posttest. Subsequently, however, this higher level of QNC facilitated children’s understanding of what was taught during regular mathematics instruction in school.

Path analytical results supported our hypothesis that long-term transfer effects on mathematics school achievement are mediated by prior gains in quantity–number competencies, a finding that further adds to the existing evidence on the crucial role of quantity–number competencies as a prerequisite for subsequent mathematics achievement.

Our findings are particularly encouraging regarding their practical implications. The results were obtained in a regular school setting, with the teacher instruction requiring only 4 h, and the entire intervention consisting of just 12 sessions. Given this minimal investment, the long-term effects on mathematical school performance of at-risk children, observable even after 15 months, are quite remarkable. According to teacher feedback, the highly standardized approach makes the program easy to implement and follow, i.e., to maintain treatment fidelity. Deviations from the program guidelines were reported only in terms of the number of weekly sessions, resulting in some groups taking longer to complete the 12 training sessions than initially planned (up to 10 weeks instead of 6).

Considering the consistently positive findings on the effectiveness of QNL training, it may be valuable to evaluate the training in future studies not as a secondary preventive measure (targeting selected at-risk children), but as a primary preventive measure by implementing QNL instruction at the beginning of first grade, prior to the regular mathematics curriculum. This approach might be even more efficient in ensuring foundational skills in mathematics at the start of first grade, thereby reducing the number of children who lack the prerequisites to benefit from subsequent conventional mathematics instruction.

One limitation of this study is that it was not conducted as a randomized controlled trial (RCT), but rather as quasi-experimental research. The assignment of participants to the experimental conditions was partially based on non-random factors, particularly to avoid diffusion effects, which introduces potential selection bias. This approach, while valuable, may not offer the same level of robustness in controlling for confounding variables as an RCT. Additionally, the relatively small sample size is a constraint, as effects observed in smaller studies often tend to diminish in larger-scale research.

Another limitation is that the models with random slopes did not achieve convergence. Thus, it cannot be entirely ruled out that the parameter estimates might be biased due to unaccounted variability in the slopes across groups. The convergence issues may be due to the relatively small number of groups (*n* = 22) and number of students per group (three to seven), since insufficient sample sizes can lead to instability in estimating parameters (Maas and Hox, 2005; Hox, 2010). Given these limitations, however, it should be noted that significant short-and long-term training effects (including both specific and transfer effects) were also confirmed in the path analyses conducted to test the mediator hypothesis, demonstrating that the fixed effects remained robust across different analytical methods. Future studies with larger sample sizes and more level-2 units could provide more reliable and nuanced insights. Finally, we considered only a limited number of control variables in our study. Future research should also examine whether the observed intervention effects are equally effective for all children or whether they are moderated by third variables such as motivational factors or cognitive influences like working memory and language proficiency.

In sum, our findings provide evidence for the long-term effectiveness of a strictly theory-based training program for the early prevention of developmental dyscalculia. Notably, this success is achieved with remarkably little effort, both in terms of teacher instruction and the total number of sessions. Additionally, these encouraging results support the QNL model’s view that dyscalculia is not due to an immutable defective number module. Instead, it supports the more optimistic notion of developmental dyscalculia as a developmental delay in foundational skills, which can be effectively addressed through targeted early prevention measures.

## Data availability statement

The raw data supporting the conclusions of this article will be made available by the authors, without undue reservation.

## Ethics statement

The studies involving humans were approved by the Ethics Committee of the Ludwigsburg University of Education. The studies were conducted in accordance with the local legislation and institutional requirements. Written informed consent for participation in this study was provided by the participants’ legal guardians/next of kin.

## Author contributions

ME: Conceptualization, Funding acquisition, Methodology, Writing – original draft, Writing – review & editing, Supervision, Formal analysis. DS: Investigation, Project administration, Formal analysis, Writing – review & editing. LN: Writing – review & editing. KK: Writing – original draft, Writing – review & editing.

## Footnotes

^1^For instance, level 1: reciting the number-word sequence forwards and backwards, naming preceding and subsequent numbers; level 2: linking number words to corresponding quantities, comparing numbers in terms of magnitude; level 3: determining numerical differences between numbers.

^2^Although different software packages typically yield comparable results, they differ substantially in terms of convergence rates (McCoach et al., 2018).

^3^Preliminary path analyses without the mediator indicated significant direct transfer effects of QNL training on later mathematics achievement (Model 1: β = 0.32; *p* < 0.001, Model 2: β = 0.28; *p* = 0.002).
