# Supplementary material for: From developmental theory to effective training: long-term and transfer effects of promoting the quantity–to–number word linkage in first-graders at risk for mathematical difficulties
Source: Front Psychol. 2024 Aug 20;15:1380036. doi: 10.3389/fpsyg.2024.1380036 (PMC11368870; doi:10.3389/fpsyg.2024.1380036)
Supplement: Supplementary file 1 [file Table_1.docx]

Supplementary Material 1: Detailed Descriptions of Interventions Targeting Foundational Mathematical Skills

# Overview

This supplement provides detailed descriptions of various studies, summarized in Table 1 of the manuscript, that evaluated intervention programs designed to enhance foundational mathematical skills comparable to the quantity-number competencies outlined in the QNL Model. These interventions range from early math programs in kindergarten to computer-based programs for at-risk children in the first grade. The following sections present the evaluated intervention programs, detailing their structure, specific aims, and underlying theoretical frameworks, with a particular focus on long-term and transfer-effects on subsequent mathematical abilities not targeted by the intervention. Furthermore, we analyze how the trained skills align with the QNL Model. We also discuss the findings of the evaluations and consider the methodological limitations of these studies.

## Number Worlds and others based on the theory by Case and Griffin

One training program that has been evaluated in these studies is the kindergarten program *Number Worlds* (Griffin, Case & Capodilupo, 1995). The aim of this program is to teach children a set of understandings that will form a knowledge network to help them grasp mathematical concepts (the “central conceptual structure of number”), that are described in the Central Conceptual Structure theory by Case and Griffin (1990). These understandings entail competencies from all three QNL levels, e.g. numbers have magnitude (level 2), numbers have fixed positions in the counting sequence (level 1), numbers that come later in the sequence indicate larger quantities (level 2), each counting number up in the sequence corresponds precisely to an increase of one unit in the size of a set (level 3). Later, children have to connect this so-called “integrated knowledge network” to formal symbols. As Griffin (2004) states, this first “enables children to make sense of a broad range of quantitative problems across contexts and to answer questions, for example, about two times on a clock (Which is longer?), two positions on a path (Which is farther?), and two sets of coins (Which is worth more?)”, connecting knowledge of counting and knowledge of quantity, see level 2 in QNL model. Second, it provides the foundation on which children’s learning of more complex number concepts, such as those involving double-digit numbers, is built” (p. 174, Griffin, 2004). Evidence for the effectiveness of this 30-week training program was found in a study by Griffin et al. (1995) with three groups of kindergarten students. While one group of children was trained with Number Worlds, a second group received an alternative mathematics intervention and a third one participated in phonological training. Four weeks after training, the Number Worlds group reached higher scores on addition and subtraction problems than both other groups. However, no (long-term) transfer effects on subsequent curriculum-based mathematics achievement in school were investigated in that study.

Sterner, Wolff, and Helenius (2019) conducted a study to investigate the effectiveness of another early math intervention program for preschoolers that is also based on the theory by Case and Griffin. The program lasted 30 min daily for ten weeks and aimed to improve the children’s number sense by teaching them to use the number word sequence flexibly (level 1 in QNL model), integrating multiple representations of number relating to both continuous quantities, numerosity, and order relations (level 2) and to decompose numbers using different representations and collective reasoning (level 3). The experimental group showed a significantly larger growth in number sense between the pre- and the post-test compared to the control group, which received regular mathematics teaching and an equally structured non-mathematical intervention program. However, the authors found no direct effect of the intervention on math skills at the follow-up test nine months later (Grade 1). They report indirect effects via the numerical post-test. It is worth mentioning that only the teachers of the experimental group had received supervision throughout the entire training period, so it cannot be ruled out that training effects were primarily due to teacher supervision rather than the math intervention alone.

## Additional Early Mathematics

The training program *Additional Early Mathematics* (Van Luit & van de Rijt, 1998) is based on Piaget’s theory of children’s cognitive development (Piaget & Szeminska, 1952) and was created to foster the mathematical development of four- to seven-year-olds with weak numerical skills. Over the course of 26 sessions with “real (daily) life contexts”, children received instruction in quantity comparison, the use of number words, the one-to-one correspondence between the single items of different sets, counting skills and number seriation, i.e. the quantitative order of ascending and decreasing numbers (all level 1 and 2 in QNL model), as well as in number relations (level 3). After intervention, the participants of the training had closed the gap between their performance and age-related norms in the *Utrecht Early Numeracy Test* (van Luit & Van de Rijt, 1998). Both short- and long-term effects (the latter seven months after training) were reported; however, there was no investigation of long-term transfer effects on mathematical school achievement beyond the skills that were the focus of training. In another study by van Luit and Schopman (2000), transfer effects were analyzed, but while specific effects on the trained skills were replicated, no transfer effect to new mathematical problems was found.

## The Number Race (computer-based)

Another program, *The Number Race*, that is computerized and based on the Triple Code Model (Dehaene, 1992) was first evaluated in a study by Wilson et al. (2006). Seven to nine year old children with mathematical difficulties were trained on the five-week computer program. Authors reported specific training effects on core number sense tasks. However, due to the lack of a control group, it remains unclear to what extent factors such as normal development, repeated testing, attention bias, and regression to the mean might have influenced the short-term group improvements. In a follow-up study on the effectiveness of The Number Race, Wilson et al. (2009) included a control group, in order to avoid these problems of interpretation. Both groups of kindergarten children received two trainings, The Number Race and a kindergarten reading package, but they passed these trainings in reverse order. For both groups short-term improvements in number sense were found subsequent to The Number Race training. However, neither effects on tasks that were not related to the contents of the math training (i.e. transfer tasks) nor long-term effects were investigated in this study. In a third study, Räsänen et al. (2009) evaluated The Number Race in two training groups of children with low numeracy skills and a control group with normal numerical achievement were included. One of the training groups used The Number Race, while the second group was trained to match small sets of objects to numbers (Graphogame-Math). Both training groups displayed higher gains in number comparison than did the typically performing control group. However, no improvements were found in verbal counting, object counting, or arithmetic, that is, there were no transfer effects on mathematical skills that were not actually the focus of training during the intervention. Long-term effects were not reported. A recent study, that investigated the new version of the program, could not replicate any short-term effects of the program on the math competencies of low-performing first graders in comparison to ordinary teacher instruction (Hellstrand, Korhonen, Linnanmäki & Aunio, 2020).

Obersteiner et al. (2013) used The Number Race as a basis, developed it further to an approximate and an exact game version and trained 147 (non-risk) first grade children using three different training conditions. The focus of the three interventions was on establishing (1) *exact* mental representations of numbers, (2) *approximate* mental representations of numbers, or (3) a *combination* of the two, respectively, targeting on level 1 and 2 in QNL model. Pre- to posttest gains in numerical performance were compared to a control group that received a language training (group 4). As indicated by a comparison of the three intervention groups, training effects were specific, that is, students’ performance improved on tasks that were part of the particular training. Moreover, intervention groups showed higher posttest scores than the control group in a test of basic numerical skills, including basic addition and subtraction tasks. However, transfer effects on mathematical abilities beyond basic addition and subtraction were not investigated. Further, since no follow-up assessment was included, and the study did not distinguish between normal achievers and children who are at-risk for developmental dyscalculia, results do not provide information about the preventive potential of theses interventions.

## Numerical Board Game

Siegler and Ramani (2008) presented the effects of the so-called *Numerical Board Game* played by four- to five-year-olds. This training program had children stride up and simultaneously recite numerals (up to 10) that were displayed in ten fields from left to right (level 1 in QNL model). In the short term, children that were trained using this approach reached higher scores in a number-line task than a control group that played a non-numerical version of the game. Whyte and Bull (2008) used this game to train three-year-olds and compared their achievement to a group of children who received a non-linear version of the game (comparing, estimating, and assigning groups of apples to their corresponding verbal and Arabic numerals; level 1 and 2 in QNL model) and to a third group that received a non-numerical training. Significant effects were found for both of the numerical training conditions, which outperformed the non-numerical condition in counting and numeral knowledge (level 1) as well as number comparison (level 2) in the short term. However, as in the other studies, neither long-term effects nor transfer effects on mathematics achievement in school were found. A recent study by Hawes and colleagues (2019), that investigated the effects of a Numerical Board Game intervention similar to that of Siegler and Ramani, in comparison to another active control training with 4- to 6-year olds replicates only a short-term effect on Arabic numeral identification (level 1), but misses any other training-related improvements.

## Rescue Calcularis (computer-based)

Specific short-term training effects were also reported in two studies with eight- to ten-year-olds, by Kucian et al. (2011) and Käser et al. (2013) for the training program *Rescue Calcularis*. This 5-week computer training program is based on the assumption that dyscalculia is a brain disorder that leads to fundamental deficits in the representation of quantities and the internal mental number line (von Aster and Shalev (2007). Therefore, the program aims to improve the construction and access to the mental number line. During 25 computer sessions (15-20 min daily for five weeks), children with dyscalculia learned to assign numerals and estimated quantities (level 1 and 2 in QNL model), or results of addition and subtraction tasks to the appropriate point on a number line going up to 100 (level 3). Immediately after training, children by Kucian and collegues showed similar gains as trained children without dyscalculia in the tasks in which they were trained. However, in the absence of a control group (without or with alternative training), these effects might well be attributed to a repeated testing bias or to developmental gains not caused by the training. Finally, in the study by Käser and collegues the trained group demonstrated larger increases as the non-trained children with dyscalculia in addition and subtraction (level 3 in QNL model), but not in number line task, magnitude comparison and estimation of dots (all level 2). In both studies long-term effects to a control group were not addressed.

## Emboddied training (computer-based)

Fischer et al. (2011) as well as Link et al. (2013) conducted a computer-based training with kindergarten children and first-graders, respectively. The authors found that embedding a (sensorimotor) spatial component (“walking the number line”) led to higher training gains in counting skills (level 1), small (0-10 scale) but not larger (0-20) number line (level 2) and reaction times, respectively, compared to a number comparison training or a training working on the very same tasks without this component. However, as in other studies, neither long-term effects nor transfer effects on a comprehensive test of mathematical school achievement were investigated. Furthermore, it is not clear why Fischer and colleagues excluded children from the final data analyses if they performed 1.5 standard deviations slower than the other participants on the pretest.

## Stimulation of the approximate number system (computer-based)

Hyde et al. (2014) investigated whether stimulation of the *approximate number system* (ANS) in first-graders enhances their performance of symbolic arithmetic. As many other researchers, authors assume that ANS is an innate primitive cognitive system shared by humans of all ages but is impaired in developmental dyscalculia. While practicing specific tasks to foster approximate numerical quantities that can assigned to level 2 and 3 in QNL model, children performed better on symbolic arithmetic problems administered from time to time than their counterparts who practiced non-numerical tasks. However, transfer and long-term effects were not investigated.

## Galaxy Math Program

Fuchs et al. (2013) compared the effectiveness of two versions of a number knowledge tutoring, the so-called *Galaxy Math Program* (Fuchs, Fuchs, & Bryant, 2010; as cited in Fuchs et al., 2013), for first grade at-risk children. This program relies on tutor instruction, guided activities and math games to promote counting numbers (level 1 in QNL model), understanding the basics of arithmetics (level 2 and 3), and utilizing manipulatives and tools for arithmetics. The authors found short-term effects on arithmetical skills for both a speeded and a non-speeded version of the training. In a further study, Fuchs et al. (2014) compared the effectiveness of two different classroom interventions at the end of second grade. Both interventions had specific effects on the particular domains that were in the focus of the training, that is, a calculation training improved students’ calculation skills while a training of word problems improved word problem outcomes. Furthermore, children who had received the calculation training were able to generalize beyond particular calculation problems that were part of the intervention. Finally, the word problem intervention (but not the calculation intervention) entailed a transfer effect on pre-algebraic knowledge. Unfortunately, no follow-up assessment was conducted to investigate the long-term durability of these effects.

## Counting and Number Comparison Games (computer-based)

Finally, Praet and Desoete (2014) conducted a training study with three groups of kindergarten children in a pre–posttest–follow-up design. The first group had to play *serious comparison games* (level 2 in QNL model), while a second group played *serious counting games* (level 1), including tasks on adding and subtracting quantities (level 3). Training group 2 showed larger pre-posttest gains in arithmetics than training group 1, which in turn significantly outperformed the control group without numerical training activities. In contrast to the vast majority of studies in the field the durability of effects was investigated a few months after training. For both training groups long-term effects on number knowledge were confirmed while only the second training had a significant long-term effect on mental arithmetics (e.g. ”16-12=__”). As in other studies, a comprehensive test of mathematical school achievement was not conducted, that is, results do not provide evidence for transfer effects on math performance in school.

# References

Case, R. & Griffin, S. (1990). Child cognitive development: The role of central conceptual structures in the development of scientific and social thought. In E. A. Hauert (Ed.), *Developmental psychology: Cognitive, perceptuomotor, and neurological perspectives* (pp. 193–230). North-Holland: Elsevier.

Dehaene, S. (1992). Varieties of numerical abilities. *Cognition, 44*, 1-42.

Fischer, U., Moeller, K., Bientzle, M., Cress, U. & Nuerk, H.-C. (2011). Sensori-motor spatial training of number magnitude representation. *Psychonomic Bulletin & Review, 18*, 177-183.

Fuchs, L. S., Geary, D. C., Compton, D. L., Fuchs, D., Schatschneider, C., Hamlett, C. L., DeSelms, J., Seethaler, P. M., Wilson, J., Craddock, C. F., Bryant, J. D., Luther, K., & Changas, P. (2013). Effects of first-grade number knowledge tutoring with contrasting forms of practice. *Journal of Educational Psychology, 105 (1),* 58-77.

Fuchs, L. S., Cirino, P. T., Powell, S. R., Schumacher, R. F., Marrin, S., Hamlett, C. L., Fuchs, D., Compton, D. L., & Changas, P. C. (2014). Does calculation or word-problem instruction provide a stronger route to prealgebraic knowledge? *Journal of Educational Psychology, 106 (4),* 990-1006.

Griffin, S. (2004). Building number sense with Number Worlds: a mathematics program for young children. *Early Childhood Research Quarterly, 19*, 173–180.

Griffin, S. A., Case, R. & Capodilupo, S. (1995). Teaching for understanding: The importance of central conceptual structures in the elementary school mathematics curriculum. In A. Strauss (Ed.), *Educational environments.* Norwood, N.J.: Ablex.

Hawes, Z., Cain, M., Jones, S., Thomson, N., Bailey, C., Seo, J., et al. (2019). Effects of a teacher-designed and teacher-led numerical board game intervention: a randomized controlled study with 4- to 6-year-olds. *Mind, Brain, and Education, 14*(1), 71–80. <https://doi.org/10.1111/mbe.12215>

Hellstrand, H., Korhonen, J., Linnanmäki, K., & Aunio, P. (2020). The Number Race – computer-assisted intervention for mathematically low-performing first graders. *European Journal of Special Needs Education, 35*(1), 85-99. <https://doi.org/10.1080/13488678.2019.1615792>

Hyde, D.C., Khanum, S., & Spelke, E.S. (2014). Brief non-symbolic, approximate number practice enhances subsequent exact symbolic arithmetic in children. *Cognition, 131,* 92–107.

Käser, T., Baschera, G. M., Kohn, J., Kucian, K., Richtmann, V., Grond, U., Gross, M., & von Aster, M. (2013). Design and evaluation of the computer-based training program Calcularis for enhancing numerical cognition. *Frontiers in Psychology, 4*, 489. <https://doi.org/10.3389/fpsyg.2013.00489>

Kucian, K., Loenneker, T., Dietrich, T., Martin, E., & von Aster, M. (2006). Impaired neural networks for approximate calculation in dyscalculic children: A functional MRI study. *Behavioral and Brain Functions, 2*, Article 31. <https://doi.org/10.1186/1744-9081-2-31>

Kucian, K., Grond, U., Rotzer, S., Henzi, B., Schonmann, C., Plangger, F., Galli, M., Martin, E. & von Aster, M. (2011). Mental number line training in children with developmental dyscalculia. *Neuroimage, 57*, 782-95.

Link, T., Moeller, K., Huber, S., Fischer, U., & Nuerk, H.-C. (2013). Walk the number line – An embodied training of numerical concepts. *Trends in Neuroscience and Education, 2*, 74-84.

Obersteiner, A., Reiss, K., & Ufer, S. (2013). How training on exact or approximate mental representations of number can enhance first-grade students’ basic number processing and arithmetic skills. *Learning and Instruction*, *23*, 125-135.

Piaget, J., & Szeminska, A. (1952). *Child’s conception of number.* London: Routledge & Kegan Paul.

Praet, M. & Desoete, A. (2014). Enhancing young children’s arithmetic skills through non-intensive, computerised kindergarten interventions: a randomised controlled study. *Teacher and Teacher Education, 39,* 56-65.

Räsänen, P., Salminen, J., Wilson, A. J., Aunio, P., & Dehaene, S. (2009). Computer-assisted intervention for children with low numeracy skills. *Cognitive Development, 24*, 450-472.

Siegler, R. S. & Ramani, G. B. (2008). Playing linear numerical board games promotes low-income children's numerical development. *Developmental Science, Special Issue on Mathematical Cognition, 11*, 655-661.

Sterner, G., Wolff, U., & Helenius, O. (2019). Reasoning about representations: Effects of an early math intervention. *Scandinavian Journal of Educational Research, 64(5),* 782-800.

Van Luit, J.E.H. van & Rijt, B.A.M. van de (1998). Stimulation of early mathematical competence. In M. Beishuizen, K.P.E. Gravemeijer & E.C.D.M. van Lieshout (Eds.), *The role of contexts and models in the development of mathematical strategies and procedures* (pp. 215-237). Utrecht: CD-B Press.

Van Luit, J. E. H. & Schopman, E. A. M. (2000). Improving early numeracy of young children with special educational needs. *Remedial and Special Education, 21,* 27-40.

von Aster, M. G. & Shalev, R. S. (2007). Number development and developmental dyscalculia. *Developmental Medicine & Child Neurology*, 49, 868-873.

Wilson, A. J., Dehaene, S., Dubois, O., & Fayol, M. (2009). Effects of an adaptive game intervention on accessing number sense in low-socioeconomic-status kindergarten children. *Mind, Brain and Education, 3*(4)*,* 224-234.

Wilson, A. J., Revkin, S. K., Cohen, D., Cohen, L., & Dehaene, S. (2006). An open trial assessment of "The Number Race", an adaptive computer game for remediation of dyscalculia. *Behavioral and Brain Functions, 2*(20).
