# Supplementary material for: From developmental theory to effective training: long-term and transfer effects of promoting the quantity–to–number word linkage in first-graders at risk for mathematical difficulties
Source: Front Psychol. 2024 Aug 20;15:1380036. doi: 10.3389/fpsyg.2024.1380036 (PMC11368870; doi:10.3389/fpsyg.2024.1380036)
Supplement: Supplementary file 2 [file Table_2.DOCX]

Supplementary Table 1: Results of Repeated Measures ANOVA

In non-randomized conditions, certain assumptions of statistical models, such as the absence of unobserved confounding factors, may not hold true. This concern is relevant not only for ANCOVA but also for multilevel models, where fixed effects may still be influenced by such confounders. To address this issue, and following a reviewer’s suggestion, we have included a repeated measures ANOVA for long-term specific training effects (repeated measures were not available for transfer effects). According to Lüdtke and Robitzsch (2023), RM ANOVA and ANCOVA (or multilevel models) have a bracketing function in quasi-experimental data, meaning the true effect will likely fall between the estimates produced by these methods. Therefore, we provide RM ANOVA to supplement the analyses reported in the main text.

**Table S1**

Results of Repeated Measures ANOVA on Specific Long-Term Training Effects on Quantity-Number Competencies (QNC)

| Source | SS | *df* | MS | *F* | *p* | η² |
| --- | --- | --- | --- | --- | --- | --- |
| *Between Subjects* | | | | | | |
| IQ | 1010.72 | 1 | 1010.72 | 27.78 | .000 | .212 |
| Basic Arithmetic | 110.50 | 1 | 110.50 | 3.04 | .084 | .029 |
| Group | 993.28 | 2 | 496.64 | 13.65 | .000 | .209 |
| Error | 3748.06 | 103 | 36.39 |  |  |  |
| *Within Subjects* | | | | | | |
| Time | 249.16 | 1 | 249.16 | 11.30 | .00 | .10 |
| Time * IQ | 29.29 | 1 | 29.29 | 1.33 | .25 | .01 |
| Time * Basic Arithmetic | 9.23 | 1 | 9.23 | .42 | .52 | .00 |
| Time * Group | 847.04 | 2 | 423.52 | 19.20 | .00 | .27 |
| Error (within) | 2271.68 | 103 | 22.06 |  |  |  |

*Note.* SS = Type III Sum of Squares; MS = Mean Square.

Lüdtke, O., & Robitzsch, A. (2023). ANCOVA versus change score for the analysis of two-wave data. *The Journal of Experimental Education,* 1-33.
